# A Frequency-Spatial Segmentation Network for High-Resolution Remote Sensing Images

**DOI:** 10.3390/s26196059

**Published:** 2026-09-24

**Authors:** Qiyuan Zhang, Jianshun Liu

**Affiliations:** School of Computer Science and Engineering, Sichuan University of Science and Engineering, Yibin 644000, China; 325085406209@stu.suse.edu.cn

**Keywords:** semantic segmentation, remote sensing, wavelet transform, spatial-frequency fusion, edge guidance

## Abstract

Semantic segmentation of high-resolution remote sensing images faces three major challenges in frequency-spatial feature fusion: background clutter mixed into high-frequency components, semantic discontinuities within large homogeneous regions, and loss of fine rigid boundaries caused by convolutional downsampling. Traditional dual-stream networks rely on complex attention mechanisms and directly concatenate heterogeneous features, which often leads to representation conflicts, information loss, and excessive edge smoothing. To address these issues, we propose FSOD-Net, a Frequency-Spatial Feature Decoupling Network built upon the SFFNet architecture. The feature mapping stage is reconstructed to incorporate physical edge priors and frequency-domain attention-based denoising. Specifically, a Spatial Attention Wavelet Transform Feature Decomposer (SA-WTFD) is introduced in the frequency branch to adaptively suppress high-frequency background clutter and enhance discriminative features. In the spatial global branch, a lightweight pyramid pooling module (L-PPM) captures macro-contextual information to fill semantic gaps within areal objects. In the local branch, a fixed Laplacian operator is embedded as an explicit physical edge prior to preserving micro-scale rigid boundaries during downsampling. These three modules operate along independent paths, enabling decoupled yet collaborative multi-domain feature learning. Extensive experiments on the ISPRS Vaihingen and Potsdam datasets demonstrate that FSOD-Net achieves mean Intersection over Union (mIoU) scores of 84.24±0.11% and 86.79±0.09%, respectively, indicating its potential in segmentation accuracy and generalization.

## 1. Introduction

With the continuous advancement of aerospace and sensor technologies, high-resolution remote sensing imagery (HRRSI) can be readily acquired, providing detailed observations of the Earth’s surface across diverse environments, ranging from complex urban scenes to natural ecosystems [1]. Semantic segmentation, as a fundamental task in remote sensing image interpretation, aims to assign pixel-level semantic labels to ground objects, which is essential for geographic information systems (GISs), resource management, environmental monitoring, and crisis management. The ever-increasing volume of high-resolution data has created a practical demand [2,3] for the automated processing and value extraction of large-scale imagery. However, the complex spectral and spatial information embedded in HRRSI, while offering rich data support for land cover interpretation, also poses substantial challenges [4,5] for high-precision feature extraction. In particular, remote sensing images are characterized by macro-scale overhead perspectives, substantial variations in object scales, highly complex backgrounds, and severe illumination and shadow interference. Consequently, suppressing redundant background interference and extracting discriminative semantic features of multi-scale ground objects remains a key challenge in computer vision and remote sensing.

Leveraging its broad coverage and high efficiency, automated remote sensing monitoring has gradually supplanted traditional manual surveys as the primary means [6] for regional observation. In early research, image segmentation relied heavily on manually designed feature extraction operators (e.g., SIFT and HOG); however, these methods exhibit limited generalization capability and are sensitive to environmental noise. With the rapid advancement of deep learning, convolutional neural networks (CNNs)—exemplified by fully convolutional networks (FCNs) and U-Net—have become the mainstream paradigm for remote sensing image segmentation due to their powerful local feature representation capabilities. Subsequent studies have alleviated, to some extent, the difficulties caused by varying object scales through mechanisms such as dilated convolution and spatial pyramid pooling (SPP). Nevertheless, due to the inherent local inductive bias of standard convolutions, CNNs still suffer from intrinsic limitations [7] in capturing long-range contextual dependencies. Given the complex spatial distribution of landforms, significant variations in object scales, and class imbalance, networks relying solely on local receptive fields remain inadequate for fine-grained multi-class segmentation [8,9].

To overcome the limitations of local receptive fields, recent studies have introduced Transformer architectures by serializing images to capture global contextual information, thereby improving the segmentation accuracy of macro-level semantics. Nevertheless, current models based on global self-attention still face three major limitations in practical remote sensing applications:

1. First, the direct coupling of heterogeneous features leads to semantic loss. Traditional dual-stream networks usually lack explicit decoupling mechanisms when fusing spatial-domain context with frequency-domain edge information. Simply concatenating these heterogeneous features or adding them pixel-wise can give rise to conflicts in the feature representation space and result in information loss, making it difficult to fully exploit the advantages of multi-domain collaboration across frequency and spatial domains.
2. Second, an inherent contradiction exists between frequency-domain denoising and the preservation of physical edges. The high-frequency subbands extracted via wavelet transform contain genuine physical edges, yet inevitably preserve a considerable amount of high-frequency background clutter. In remote sensing scenarios with complex illumination interference, such as shadow occlusion and vegetation textures, directly incorporating high-frequency features without appropriate selection may fail to guide segmentation and can even lead to semantic misclassification due to the intrusion of background clutter.
3. Third, macro- and micro-feature extraction faces coupling conflicts. To capture multi-scale landforms, existing models rely heavily on deep convolutions or attention-based pooling operations. Although this mechanism can extract global context, it inevitably causes excessive smoothing of features along the spatial dimension, thereby exacerbating the loss of rigid boundary information for fine linear features such as roads and building outlines.

Figure 1 illustrates how these limitations result in semantic fragmentation and boundary degradation in complex remote sensing scenes.

To address the aforementioned limitations, this paper departs from the design paradigm that relies on stacking complex attention mechanisms. Building upon the SFFNet architecture, we restructure the feature mapping stage at the module level and propose a Frequency-Spatial Feature Decoupling Network, termed FSOD-Net. The main contributions of this paper are as follows:

1. Proposal of FSOD-Net: To address the lack of selective coupling in traditional dual-stream networks when fusing heterogeneous features, we orthogonally decompose the feature extraction task into three dimensions—frequency-domain purification, global context aggregation, and micro-boundary preservation—thereby alleviating semantic conflicts and information loss caused by direct interaction of heterogeneous features.
2. Proposal of SA-WTFD: In the frequency-domain feature extraction branch, we construct a spatial attention mask to adaptively purify the high-frequency subbands after Haar wavelet decomposition. This mechanism serves to suppress noise interference while effectively preserving true geometric edge features.
3. Proposal of Dual-Scale Spatial Perception Collaborative Mechanism (L-PPM and EGA-Local): In spatial feature extraction, we employ a lightweight pyramid pooling module to aggregate macro-level contextual semantics, thereby filling semantic holes within large-scale homogeneous regions. Simultaneously, we embed a fixed Laplacian operator into the local branch as an explicit physical edge prior to alleviate the degradation of micro-level rigid boundaries caused by deep convolutional downsampling.

## 2. Related Work

### 2.1. Remote Sensing Image Semantic Segmentation

One of the core challenges in the semantic segmentation of high-resolution remote sensing images (HRRSIs) lies in the precise preservation of boundaries for complex areal features and minute objects [10,11,12]. Although early methods relied heavily on low-level feature extraction [13,14], deep learning architectures—represented by convolutional neural networks (CNNs)—have emerged as the most efficient and mainstream paradigm in this field due to their robust feature representation capabilities [15,16,17]. Particularly in identifying minute, densely distributed, and scale-variable features, deep CNN architectures have demonstrated advantages [18,19,20]. However, traditional CNNs are constrained by the local receptive fields of standard convolutions, making it difficult to fully capture global contextual information [21]. Furthermore, single-stream CNNs ignore long-range dependencies across multimodal feature maps, failing to effectively learn discriminative ensemble representations for segmentation [22]. This limitation in local perception causes models to be highly susceptible to semantic fragmentation and voids within large-scale homogeneous regions when processing HRRSIs.

To break through this bottleneck and enhance context awareness, recent studies have widely introduced multiscale aggregation mechanisms and Transformer-based global self-attention architectures [23,24,25]. Nevertheless, when comprehensively describing the spatial relationships of complex remote sensing objects, these existing models—despite aiming to expand the receptive field—still expose notable limitations [26,27]. In complex urban scenes, the blind implicit feature coupling conducted to capture global semantics easily provokes internal information attrition and feature conflicts. While this mechanism can fill voids within homogeneous regions to some extent, it inevitably leads to excessive spatial feature smoothing, thereby deteriorating the microscopic rigid boundaries of fine linear features (e.g., roads and building contours).

Therefore, the key to overcoming the current accuracy bottleneck in high-resolution remote sensing semantic segmentation lies in breaking the direct coupling of heterogeneous features while preserving the geometric boundaries of ground objects with high fidelity. To address this issue of microscopic boundary loss, we introduce the EGA-Local into the spatial local branch. By providing explicit physical edge priors, this mechanism counteracts the rigid boundary erosion induced by continuous downsampling in deep networks, thereby achieving extraction and preservation of fine linear contours.

### 2.2. Multiscale Context Aggregation

In the semantic segmentation of remote sensing images, macroscopic contextual information is crucial for eliminating semantic fragmentation within large-scale homogeneous regions, such as extensive buildings, water bodies, and vegetation. The classic PSPNet, employing spatial pyramid pooling (SPP), first demonstrated the effectiveness of multiscale receptive fields in complex scene parsing. Subsequently, the DeepLab series of models [28] further proposed Atrous Spatial Pyramid Pooling (ASPP). By introducing dilated (atrous) convolutions, this architecture effectively expands the receptive field without sacrificing the spatial resolution of feature maps, thereby further enhancing the feature consistency of areal objects.

However, traditional context aggregation modules are typically accompanied by large parameter counts and high computational complexity. More importantly, when fusing multiscale features, these methods often employ direct channel-wise concatenation or pixel-wise addition. This not only causes excessive spatial feature smoothing but also easily introduces substantial background noise [29], which degrades the reliability of multiscale prediction results [30]. In high-resolution remote sensing scenarios, such oversmoothing effects obscure weak high-frequency features and exacerbate the dispersion and loss of object boundary characteristics.

Therefore, considering the unique characteristics of remote sensing data, there is an urgent need to refine multiscale fusion strategies by designing an aggregation mechanism that captures long-range contextual dependencies, maintains a lightweight architecture, and avoids interference with local high-frequency details [31,32]. Motivated by this, we propose a L-PPM. By employing decoupled parallel pooling branches with a gated residual injection strategy, this module achieves the extraction and integration of global macro-level semantics while minimizing computational cost.

### 2.3. Frequency-Domain Feature Decomposition and Spatial Attention

The effective fusion of spatial and frequency information has been demonstrated as a crucial approach for enhancing the representational capability of visual models and addressing the complex segmentation challenges of high-resolution remote sensing imagery (HRRSI) [33,34]. HRRSI typically contains rich textural details and complex high-frequency noise. Traditional convolutional neural networks (CNNs), which primarily extract features from a single spatial domain, are inherently constrained by their local inductive bias. This makes them prone to misinterpreting high-frequency structural features as irrelevant background noise, thereby leading to a noticeable degradation in discriminative ability at microscopic boundaries.

To overcome the limitations of single-domain perception, frequency-domain analysis has gradually been introduced into deep feature extraction. Compared with the traditional Fourier transform, the discrete wavelet transform (DWT)—with its time-frequency localization property—can orthogonally decompose a target signal into low-frequency approximation components and multi-scale, multi-directional high-frequency detail components. This has demonstrated clear advantages in mitigating information loss caused by downsampling [35,36]. However, most existing studies treat DWT merely as a static substitute for pooling, without fully exploiting the discriminative information embedded in the high-frequency subbands. In complex urban scenes with interwoven ground objects, indiscriminately fusing all high-frequency subbands tends to introduce background clutter and cause feature redundancy. Therefore, how to efficiently coordinate spatial and frequency-domain information and establish an adaptive filtering mechanism for high-frequency noise remains a key issue that has yet to be resolved [37].

To address the aforementioned limitations, we propose the SA-WTFD. Grounded in the collaborative design philosophy of “frequency-domain decoupling and spatial focusing,” SA-WTFD introduces an adaptive spatial attention mask to dynamically purify the high-frequency subbands after wavelet decomposition. SA-WTFD effectively suppresses high-frequency background clutter, while preserving the geometric structures and microscopic physical boundaries that are important for subsequent segmentation tasks.

## 3. Methodology

In this section, we first outline the overall network architecture of FSOD-Net and its core design philosophy of three-dimensional orthogonal decoupling in the frequency-spatial domain. Subsequently, we detail three key functional modules introduced in the feature mapping stage: L-PPM aggregates global contextual information, SA-WTFD suppresses frequency-domain noise and enhances high-frequency representations, while EGA-Local preserves rigid boundaries at the microscopic level. Building on this, we further describe the Multiscale Dual-Representation Alignment Filter (MDAF) [38], which bridges the semantic gap between spatial and frequency-domain features. Finally, we present the joint loss function used for model optimization and its mathematical formulation.

### 3.1. Network Architecture

To validate the effectiveness of the proposed decoupling mechanism, we construct a basic feature extractor using ConvNext-Tiny as the backbone network. Meanwhile, to exploit multi-scale spatial semantics and high-frequency details from remote sensing imagery while preserving computational efficiency, we adopt SFFNet as the baseline and perform a module-level restructuring of its feature mapping stage (FMS). Specifically, to address the segmentation bottlenecks of high-resolution remote sensing data, we replace the original three branches—global, local, and frequency—with L-PPM, EGA-Local, and SA-WTFD, respectively, thereby achieving a physical-level reconstruction and network-level upgrade of the feature mapping network.

As shown in Figure 2, the overall architecture of FSOD-Net comprises three core stages: the Spatial Feature Extraction Stage, the FMS, and the Cross-Domain Fusion and Segmentation Stage. To concentrate the research focus on the frequency-spatial three-dimensional orthogonal decoupling within the FMS, the cross-domain fusion stage adopts the Multiscale Dual-Representation Alignment Filter (MDAF) from prior work [38].

#### 3.1.1. Spatial Feature Extraction Stage

The core task of this stage is to extract multi-level spatial semantic features from the input raw high-resolution remote sensing imagery. We select ConvNext-Tiny as the backbone network. Given an input image, the backbone outputs four hierarchical features with different spatial resolutions and channel dimensions, denoted as $x_{1}$, $x_{2}$, $x_{3}$, and $x_{4}$, respectively.

To aggregate multiscale semantics, we select the three deeper features ($x_{2}$, $x_{3}$, and $x_{4}$). We first unify their channel dimensions via 1 × 1 convolutions, then upsample them using bicubic interpolation to precisely align the spatial resolutions. The aligned features are then concatenated along the channel dimension to generate a multi-scale contextual feature map, which is fed into the subsequent FMS for further decoupling.

#### 3.1.2. Feature Mapping Stage

Traditional feature extraction mechanisms rely heavily on computationally expensive spatial self-attention models, which not only lead to excessive smoothing of local features but also introduce environmental high-frequency noise. To achieve deep feature decoupling and purification, we restructure the FMS by orthogonally splitting the cascaded baseline features into three independent parallel branches. These three branches are illustrated in detail in Figure 3:

- Spatial Local Branch: EGA-Local
  In complex remote sensing scenarios, the geometric contours of rigid objects—such as buildings and roads—are susceptible to distortion and blurring during the progressive downsampling operations of deep networks. To address this issue, the proposed branch abandons the conventional implicit parameter learning paradigm and introduces a non-parametric Laplacian operator as an explicit physical edge prior. The Laplacian kernel itself introduces no learnable parameters; it extracts and preserves fine-grained rigid boundaries directly from the feature maps. The extracted edge priors are then aligned to the target feature space via a lightweight projection layer that contains only a small number of learnable parameters. Coupled with subsequent feature preservation and gated residual injection, this branch ultimately produces local spatial features with high boundary fidelity;

- Frequency-Domain Decoupling Branch: SA-WTFD
  To handle the complex backgrounds and severe illumination variations in high-resolution remote sensing imagery, the proposed branch first employs the Haar discrete wavelet transform (DWT) to decompose the baseline spatial features into low-frequency semantic components and high-frequency detail and edge components. To mitigate the inherent limitation of standard DWT—where the extracted high-frequency subbands are inevitably contaminated by background clutter—an adaptive spatial attention mask is cascaded after high-frequency extraction. This mask dynamically suppresses environmental high-frequency noise, thereby preserving the true geometric and physical edges. Ultimately, the branch simultaneously outputs low-frequency semantic features and high-frequency detail features that have been dynamically denoised;
- Spatial Global Branch: L-PPM
  To alleviate the receptive field limitation caused by removing the computationally expensive global window attention mechanism, we deploy L-PPM. Through parallel multi-scale adaptive average pooling, this module aggregates global contextual semantics with minimal computational cost, filling “semantic discontinuities” and holes within large contiguous land covers, and ultimately outputs context-aware features with global long-range perception.

#### 3.1.3. Cross-Domain Fusion and Segmentation Stage

After obtaining the four decoupled feature representations—$F_{m}$, $F_{l}$, $F_{h}$, and $F_{g}$—the network proceeds to the cross-domain fusion stage. To bridge the semantic gap between frequency-domain and spatial-domain features, the Multiscale Dual-Representation Alignment Filter (MDAF) [38] is employed. As illustrated in Figure 2, MDAF is instantiated as two parallel modules: ${\mathrm{MDAF}}_{L}$ for low-frequency and global feature fusion, and ${\mathrm{MDAF}}_{H}$ for high-frequency and local feature fusion.

The low-frequency features $F_{l}$, which encapsulate homogeneous background semantics, and the global context features $F_{g}$ extracted by L-PPM are fed into the ${\mathrm{MDAF}}_{L}$ module [38]. This module performs cross-modal interaction and fusion at the macro-semantic level, aligning the complementary representations from the frequency and spatial domains.

The high-frequency features $F_{h}$, rich in edge details, and the local rigid features $F_{m}$ extracted by EGA-Local are jointly fed into the ${\mathrm{MDAF}}_{H}$ module [38]. Within this module, fine-grained boundary alignment is achieved through a cross-attention mechanism, enabling effective integration of complementary edge and structural information.

The two fused features are then concatenated and integrated via a Weighted Fusion Block (WF), followed by a skip connection to the high-resolution shallow feature $x_{1}$ from the backbone. The resulting hybrid features are finally fed into the segmentation head to produce the pixel-level semantic segmentation output.

FSOD-Net adopts an encoder–decoder architecture and features a three-branch decoupling mechanism during the feature extraction stage (Figure 2). Given an input image, the network first applies the Haar wavelet transform to decompose it into frequency streams, yielding low-frequency features $F_{l}$ and high-frequency features $F_{h}$. Simultaneously, a convolutional network is used to extract spatial features. Unlike traditional approaches, we explicitly divide the spatial stream into two sub-paths—“global” and “local”—to obtain spatial global features $F_{g}$ and multi-scale local features $F_{m}$, respectively. At this point, the network has completed feature decoupling, providing four mutually independent feature representations ($F_{g}$, $F_{l}$, $F_{h}$, $F_{m}$) for subsequent processing. The overall feature extraction stage is driven by three core modules: SA-WTFD processes the frequency features ($F_{l}$ and $F_{h}$) and suppresses high-frequency clutter in the frequency domain; L-PPM, which operates on $F_{g}$ for context aggregation in the spatial global domain; and EGA-Local, which handles $F_{m}$ for microscopic edge preservation in the spatial local domain.

### 3.2. Introduction to Core Modules

#### 3.2.1. L-PPM

To achieve fast inference, we remove the global window attention mechanism from the original network, which has quadratic computational complexity $O(N^{2})$. However, this modification weakens the network’s macro perspective, leading to “semantic holes” within categories when segmenting large contiguous land covers (e.g., large factories or open water bodies). To address this, we introduce L-PPM to restore multi-scale context-aware capability with low computational cost.

The input feature map is first downsampled to obtain the base feature $X_{\mathrm{base}}\in R^{C\times H^{\prime }\times W^{\prime }}$. To capture semantic information across scales—from coarse to fine—L-PPM employs three parallel adaptive average pooling layers, which compress the spatial resolutions to 1×1, 2×2, and 3×3, respectively. The process is formulated as follows:

(1) $$P_{i}={\mathrm{AdaptiveAvgPool}}_{i\times i}\left(X_{\mathrm{base}}\right),\;i\in \left\{1,2,3\right\}$$

Here, $P_{i}$ denotes the feature map output by the *i*-th branch after adaptive average pooling, $X_{\mathrm{base}}$ is the input base feature map, *i* is the pooling scale parameter, and ${\mathrm{AdaptiveAvgPool}}_{i\times i}\left(\cdot \right)$ represents the operation that resizes the feature map to i×i, where i∈{1,2,3}.

The multi-scale features are then upsampled to the original spatial resolution $H^{\prime }\times W^{\prime }$ via bilinear interpolation and concatenated with $X_{\mathrm{base}}$ along the channel dimension to obtain the fused feature $C_{\mathrm{concat}}$. This process is formulated as follows:

(2) $$C_{\mathrm{concat}}=\left[X_{\mathrm{base}};\mathrm{Up}\left(P_{1}\right);\mathrm{Up}\left(P_{2}\right);\mathrm{Up}\left(P_{3}\right)\right]$$

Here, Up(·) denotes bilinear interpolation, [·;·] represents tensor concatenation, $C_{\mathrm{concat}}\in R^{C^{\prime }\times H^{\prime }\times W^{\prime }}$ is the concatenated feature tensor, and $C^{\prime }$ is the total number of channels after concatenation.

Finally, a 1×1 convolution is applied to reduce the channel dimension and fuse cross-scale information. Incorporating the zero-initialized adaptive parameter $\gamma_{\mathrm{global}}$ and the ReLU activation, the output $F_{g}$ is formulated as

(3) $$F_{g}=\mathrm{ReLU}\left(X_{\mathrm{base}}+\gamma_{\mathrm{global}}\cdot {\mathrm{Conv}}_{1\times 1}\left(C_{\mathrm{concat}}\right)\right)$$

Here, ${\mathrm{Conv}}_{1\times 1}\left(\cdot \right)$ denotes a convolution with a kernel size of 1×1, ReLU(·) is the rectified linear unit activation, $\gamma_{\mathrm{global}}$ is a learnable adaptive parameter for residual weight adjustment (initialized to 0), and $F_{g}$ represents the final macro-contextual feature output.

Through this design, L-PPM introduces only a few convolutional parameters while providing the network with a macro receptive field that covers the entire scene, thereby suppressing intra-class fragmentation within large-scale areal objects.

To alleviate the receptive field limitation caused by removing the global self-attention mechanism, the feature flow is designed to route the baseline features in parallel into three Adaptive Average Pooling (AAP) branches with different receptive field scales (spatial sizes of 1×1, 2×2, and 3×3, respectively). These branches construct a hierarchical receptive field via multi-scale pooling, capturing both global macro-level semantics and region-level contextual dependencies.

The extracted multi-scale features are then upsampled via bilinear interpolation to restore the original spatial resolution and concatenated with the baseline features along the channel dimension. Through cross-channel interaction via 1×1 convolutions and ReLU activation, the concatenated multi-scale semantics are aggregated and condensed. This parallel pyramid design circumvents the quadratic computational complexity of traditional self-attention while filling “semantic discontinuities” and voids within large contiguous areal features with minimal convolutional parameter overhead.

#### 3.2.2. SA-WTFD

High-resolution remote sensing images typically contain complex background textures (e.g., forest canopies and water ripples) along with illumination and shadow variations. Local grayscale fluctuations in these backgrounds can interfere with feature extraction, leading to prediction errors. Although the traditional wavelet transform is capable of extracting high-frequency details, when applied to complex remote sensing scenes, it may retain background clutter within the high-frequency subbands and propagate it to subsequent feature mapping stages. To address this, we propose SA-WTFD, which constructs a spatial attention mask to dynamically suppress high-frequency background noise, thereby enabling effective extraction and preservation of the physical boundary features of target objects.

Here, SA-WTFD leverages the alternating row-column filtering mechanism of the Haar wavelet transform to orthogonally decompose the input features into four subbands: the low-frequency approximation component $Y_{L}$ (LL), the horizontal high-frequency component $Y_{LH}$ (LH), the vertical high-frequency component $Y_{HL}$ (HL), and the diagonal high-frequency component $Y_{HH}$ (HH). Among these, $Y_{L}$ represents the global structure and fundamental semantics, while the other three components capture edge and texture details along their respective orientations. Subsequently, SA-WTFD concatenates these three high-frequency subbands along the channel dimension and applies a spatial attention mask to them, aiming to adaptively suppress background noise while preserving the physical edge responses of target objects.

Specifically, given the input feature *X*, we first apply the two-dimensional Haar discrete wavelet transform (2D-DWT) (Figure 4) to decompose it into a low-frequency approximation component $Y_{L}$ and three high-frequency detail components $\left\{Y_{HL},Y_{LH},Y_{HH}\right\}$ corresponding to the horizontal, vertical, and diagonal directions. This process is formulated as

(4) $$Y_{L},\;\left\{Y_{HL},Y_{LH},Y_{HH}\right\}={\mathrm{DWT}}_{\mathrm{Haar}}\left(X\right)$$

To integrate multi-directional high-frequency information, we first concatenate the three high-frequency components along the channel dimension to obtain the composite feature $Y_{H\_\mathrm{cat}}$. To suppress background noise, we then apply max pooling ($P_{\max}$) and average pooling ($P_{\mathrm{avg}}$) along the channel dimension, generating a two-dimensional spatial attention descriptor:

(5) $$P_{\max}=\max_{c}\left(Y_{H\_\mathrm{cat}}\right),\;P_{\mathrm{avg}}=\frac{1}{C}\sum_{c=1}^{C}Y_{H\_\mathrm{cat}}$$

Furthermore, we concatenate the two pooled features along the channel dimension and apply a 7×7 large-kernel convolution for cross-channel fusion and spatial dimensionality reduction. Finally, we generate the two-dimensional spatial attention mask $M_{\mathrm{spatial}}$ via the Sigmoid activation function, formulated as

(6) $$M_{\mathrm{spatial}}=\sigma \left({\mathrm{Conv}}_{7\times 7}\left([P_{\max};P_{\mathrm{avg}}]\right)\right).$$

Here, [·;·] denotes channel-wise concatenation, ${\mathrm{Conv}}_{7\times 7}$ represents a 7×7 convolution, and σ is the Sigmoid activation function. The mask values range in [0,1], indicating the probability that a spatial location corresponds to a high-frequency edge of a target object. We then perform element-wise multiplication between this mask and the composite high-frequency features to adaptively filter out environmental noise, yielding the purified feature $Y_{H\_\mathrm{clean}}$:

(7) $$Y_{H\_\mathrm{clean}}=Y_{H\_\mathrm{cat}}⊙M_{\mathrm{spatial}}$$

Finally, to preserve gradient smoothness and training stability, we introduce a learnable dynamic weight $\gamma_{\mathrm{freq}}$ with zero initialization for residual updates, obtaining the final denoised high-frequency feature $F_{h}$:

(8) $$F_{h}=Y_{H\_\mathrm{cat}}+\gamma_{\mathrm{freq}}\cdot \left(Y_{H\_\mathrm{clean}}-Y_{H\_\mathrm{cat}}\right)$$

The input features are first decomposed via a two-dimensional Haar discrete wavelet transform (2D-DWT) into a low-frequency component that captures global structural information and three high-frequency components that characterize local edge details. To suppress high-frequency clutter such as forest patchiness and water ripples within the high-frequency subbands, we design a parallel spatial mask extractor. This module applies max-pooling and average-pooling to the composite high-frequency features along the channel dimension, aggregating them into a two-dimensional spatial representation. A large-kernel convolution followed by Sigmoid activation then generates a pixel-level soft mask. This mask dynamically suppresses background responses via element-wise multiplication, preserving the high-frequency boundaries of rigid objects such as buildings and roads. Finally, we perform feature updates via a zero-initialized residual connection, yielding the purified frequency-domain features.

#### 3.2.3. EGA-Local

In remote sensing image segmentation, the boundaries of rigid objects such as buildings and roads are susceptible to distortion and blurring during the continuous downsampling process of deep networks. Traditional local attention mechanisms attempt to recover edge details via implicit parameter learning, which often introduces overfitting risks and incurs high computational cost. To this end, this stage introduces an Explicit Physical Edge Prior Extractor (EGA-Local), which directly employs the classical Laplacian operator as a non-parametric prior. The subsequent projection layer introduces only a small number of learnable parameters, and the overall architecture effectively mitigates the high computational cost typically associated with boundary learning.

The computation proceeds as follows: given an input feature map $X\in R^{C\times H\times W}$, we first define a fixed 3×3 Laplacian kernel $K_{\mathrm{Laplace}}$ to capture second-order spatial derivatives:

(9) $$K_{\mathrm{Laplace}}=\left[\begin{array}{ccc} -1 & -1 & -1\\ -1 & 8 & -1\\ -1 & -1 & -1 \end{array}\right]$$

To match the channel dimension without adding parameters, we expand this kernel along the depth dimension into a depthwise kernel with *C* channels. A grouped depthwise convolution with *C* groups is then applied to extract the raw edge responses $E_{\mathrm{raw}}$:

(10) $$E_{\mathrm{raw}}=X⊛K_{\mathrm{Laplace}}$$

Here, ⊛ denotes a depthwise convolution with *C* groups. Since the network subsequently performs downsampling with a stride of 2, and to prevent the loss of fine edge features during this reduction, we cascade a max-pooling operation aligned with the downsampling stride to preserve the edge responses $E_{\mathrm{survive}}$:

(11) $$E_{\mathrm{survive}}={\mathrm{MaxPool}}_{2\times 2}\left(E_{\mathrm{raw}}\right)$$

Subsequently, the extracted edge prior features are mapped to the target feature space through a lightweight projection layer, yielding the aligned prior features $E_{\mathrm{prior}}$. The projection layer is formulated as follows:

(12) $$E_{\mathrm{prior}}=\mathrm{GELU}\left(\mathrm{BN}\left({\mathrm{Conv}}_{1\times 1}\left(E_{\mathrm{survive}}\right)\right)\right)$$

In this formulation, ${\mathrm{Conv}}_{1\times 1}\left(\cdot \right)$ performs channel reduction and dimensional alignment, BN(·) stabilizes the feature distribution across deep layers via batch normalization, and GELU(·) introduces non-linear expressiveness to enhance the representational capacity of edge features.

To ensure convergence stability during early training, we introduce a zero-initialized learnable weight $\gamma_{\mathrm{edge}}$ to inject the edge prior into the baseline features $X_{\mathrm{base}}$ through a gated residual connection. The final local spatial features $F_{m}$ are then computed as

(13) $$F_{m}=X_{\mathrm{base}}+\gamma_{\mathrm{edge}}\cdot E_{\mathrm{prior}}$$

This architectural design renders the network equivalent to conventional downsampling in the early training stages. As optimization proceeds, the model adaptively integrates the explicit physical edge priors, thereby enhancing boundary segmentation accuracy with only marginal increases in parameter count and computational cost.

The feature flow in EGA-Local adheres to a “non-destructive extraction” principle. The input is bifurcated into two parallel streams: the main pathway passes the baseline features directly via identity mapping to preserve the underlying spatial semantics, while the auxiliary pathway applies a fixed 3×3 Laplacian depthwise kernel to compute second-order spatial derivatives, thereby extracting the physical boundaries of objects. Since the features fed into EGA-Local have already undergone multi-level convolutions and downsampling within the backbone, the high-frequency noise originally present in the image has been largely smoothed out. Thus, applying the second-order Laplacian operator in this deep feature space not only avoids the traditional pitfall of amplifying noise but also enables extraction of geometric boundary responses. To mitigate potential edge loss during downsampling, the auxiliary pathway cascades a max-pooling operation to retain high-response edge pixels. Finally, after dimensionality reduction and alignment via a projection layer, the geometric priors are modulated by the learnable gating coefficient $\gamma_{\mathrm{edge}}$ and re-injected into the main branch via a residual connection.

### 3.3. Loss Function

In remote sensing semantic segmentation, high-resolution images often exhibit class imbalance in spatial distribution. To jointly optimize pixel-wise classification accuracy and improve the model’s sensitivity to the Intersection over Union (IoU) of minority classes, we adopt a combined loss function that integrates Cross-Entropy Loss ($L_{\mathrm{ce}}$) and Dice Loss ($L_{\mathrm{dice}}$), following the baseline configuration. This joint formulation guides end-to-end backpropagation and parameter optimization, balancing pixel-level discrimination and region-level overlap.

For a given batch, let *N* denote the batch size, *H* and *W* the spatial dimensions of the feature map, and *K* the number of classes. The cross-entropy loss $L_{\mathrm{ce}}$ quantifies the pixel-wise classification error by computing the average negative log-likelihood over all spatial positions. It is formulated as

(14) $$L_{\mathrm{ce}}=-\frac{1}{N\times H\times W}\sum_{n=1}^{N}\sum_{h=1}^{H}\sum_{w=1}^{W}\sum_{k=1}^{K}y_{k}^{\left(n,h,w\right)}\log{\hat{y}}_{k}^{\left(n,h,w\right)}$$

Here, $y_{k}^{\left(n,h,w\right)}\in \left\{0,1\right\}$ denotes the one-hot ground-truth label indicating whether the pixel at spatial position (h,w) in the *n*-th image belongs to class *k*, and ${\hat{y}}_{k}^{\left(n,h,w\right)}\in \left[0,1\right]$ is the corresponding predicted probability after Softmax normalization.

To further alleviate class imbalance, Dice Loss ($L_{\mathrm{dice}}$) is introduced to regularize the network from a global region-overlap perspective, effectively mitigating the bias caused by varying object scales. It is defined as

(15) $$L_{\mathrm{dice}}=1-\frac{2}{N\cdot K}\sum_{n=1}^{N}\sum_{k=1}^{K}\frac{\sum_{h=1}^{H}\sum_{w=1}^{W}y_{k}^{\left(n,h,w\right)}{\hat{y}}_{k}^{\left(n,h,w\right)}}{\sum_{h=1}^{H}\sum_{w=1}^{W}\left({\hat{y}}_{k}^{\left(n,h,w\right)}+y_{k}^{\left(n,h,w\right)}\right)+\epsilon }$$

Here, $\epsilon =10^{-6}$ is a smoothing term that faithfully reflects the engineering implementation and prevents numerical instability, such as division by zero, induced by extreme class imbalance. The final joint loss function is then defined as the sum of the two components.

(16) $$L=L_{\mathrm{ce}}+L_{\mathrm{dice}}$$

In the above formulation, N×H×W denotes the total number of pixels in the batch, and the summations explicitly iterate over every spatial location (h,w), ensuring that the loss is computed strictly at the pixel level. By jointly optimizing the two losses, the model achieves a balance between pixel-wise classification accuracy and global structural coherence in complex urban scenes.

In constructing the joint loss, we adopt an equal-weight fusion scheme for $L_{\mathrm{ce}}$ and $L_{\mathrm{dice}}$, whose theoretical basis lies in the complementary nature of their optimization objectives. Specifically, $L_{\mathrm{ce}}$ provides fine-grained pixel-wise supervision, while $L_{\mathrm{dice}}$ evaluates the global overlap between the predicted segmentation and the ground truth. Since the Dice coefficient inherently mitigates the dominance of majority classes in gradient updates, this equal-weight coupling implicitly enforces adaptive class-balancing constraints. From the perspective of experimental objectivity, this hyperparameter-free configuration is consistent with the baseline loss settings, thus avoiding potential performance biases introduced by manual tuning.

## 4. Experiments

### 4.1. Experimental Setup

#### 4.1.1. Datasets

The ISPRS (International Society for Photogrammetry and Remote Sensing) Vaihingen and Potsdam datasets are widely used benchmark datasets in remote sensing semantic segmentation. They are frequently employed for standardized algorithm evaluation to validate model designs and ensure result comparability. Both datasets cover diverse land cover categories and complex environmental conditions, including buildings, roads, vegetation, and other typical land features, across scenarios ranging from regular urban blocks to intricate surface textures. This diversity facilitates objective assessment of model robustness and generalization under category variability, multiscale variations, and cross-domain distribution shifts. As these datasets have been established as standard testbeds in the literature, their use enables fair and rigorous comparison with existing methods. The class distributions of these two datasets are illustrated in Figure 5, and detailed descriptions of each dataset are provided below.

ISPRS Vaihingen Dataset: This dataset comprises aerial remote sensing images acquired over the Vaihingen region in Germany. It includes 33 high-resolution true orthophoto (TOP) tiles, each with an average size of 2494×2064 pixels. The data provide true orthophotos, digital surface models (DSM), and normalized digital surface models (nDSM). In terms of annotation, the dataset covers five foreground classes (impervious surfaces, buildings, low vegetation, trees, and cars) and one background class (clutter). To ensure a fair comparison with existing studies, we follow the official ISPRS benchmark split, selecting 16 images with specific IDs (1, 3, 5, 7, 11, 13, 15, 17, 21, 23, 26, 28, 30, 32, 34, 37) for training, and the remaining 17 for testing. To ensure consistency with the baseline network, the model adopts a three-channel input comprising near-infrared, red, and green (IR-R-G) bands. During preprocessing, the original images are uniformly cropped into non-overlapping tiles of 1024×1024 pixels as the fundamental input units for network loading.

ISPRS Potsdam Dataset: This dataset originates from aerial images of the Potsdam region in Germany, comprising 38 high-resolution TOP tiles with a ground sampling distance (GSD) of 5 cm and a size of 6000×6000 pixels per tile. Similar to Vaihingen, it provides true orthophotos and digital surface models (DSM), along with four multispectral bands: red, green, blue, and near-infrared (R-G-B-NIR). For data partitioning, we follow the official ISPRS training split, selecting images with IDs 2_10, 2_11, 2_12, 3_10, 3_11, 3_12, 4_10, 4_11, 4_12, 5_10, 5_11, 5_12, 6_7, 6_8, 6_9, 6_10, 6_11, 6_12, 7_7, 7_8, 7_9, 7_11, and 7_12 for training, The remaining 15 images (2_13, 2_14, 3_13, 3_14, 4_13, 4_14, 4_15, 5_13, 5_14, 5_15, 6_13, 6_14, 6_15, 7_10, and 7_13) serve as the test set. For the input channels, the model utilizes three-channel RGB (red, green, blue) images.All raw images used for training and testing were uniformly cropped into 1024×1024-pixel patches following a unified protocol. Note that the “clutter” class is excluded from the final quantitative evaluation.

#### 4.1.2. Implementation Details

All experiments were conducted on a Windows 11 Pro system using PyTorch 1.11 and deployed on a single NVIDIA RTX 4500 GPU with 24 GB of memory. To accelerate convergence and ensure optimization stability, we adopt the AdamW optimizer and introduce a cosine annealing learning rate schedule with warm restarts. Specifically, the initial learning rate is set to $6\times 10^{-4}$, with the first restart period $T_{0}=15$ epochs and a period multiplier $T_{\mathrm{mult}}=2$. Over the full 105-epoch training process, the network follows three annealing cycles of 15, 30, and 60 epochs, respectively. This periodic restart mechanism effectively modulates the gradient step size during training, facilitating escape from local minima and promoting improved generalization performance in complex high-resolution remote sensing scenarios.

For the Vaihingen and Potsdam datasets, the preprocessing and augmentation protocols are as follows. Based on the 1024×1024 base tiles obtained in the preliminary stage, the network performs online random cropping to 512×512 during training. To enrich spatial and scale diversity, we apply multi-scale random scaling with factors of {0.5,0.75,1.0,1.25,1.5}, random horizontal and vertical flipping, and random rotation. The training process is fixed at 105 epochs. No separate validation set is constructed. During inference, the network takes the full 1024×1024 tiles as input and adopts Test-Time Augmentation (TTA) with multi-scale evaluation and random flipping to enhance prediction robustness and segmentation accuracy in complex scenarios. To assess the effect of random initialization, each ablation configuration is independently trained three times with random seeds 0, 42, and 3407, and results are reported as mean ± standard deviation.

### 4.2. Evaluation Metrics

To quantitatively evaluate the proposed method on the ISPRS Vaihingen and Potsdam datasets, we adopt three standard metrics: Overall Accuracy (OA), mean F1-score (mF1), and mean Intersection over Union (mIoU).

These metrics are derived from the confusion matrix, which relies on four basic statistics: True Positives (TP), False Positives (FP), True Negatives (TN), and False Negatives (FN). The definitions and formulas for each metric are provided below.

1. (OA): Overall Accuracy (OA) measures the proportion of correctly classified pixels relative to the total number of pixels. In multi-class semantic segmentation, it is defined as the ratio of the total number of correctly classified pixels to the total number of pixels:
  (17) $$\mathrm{OA}=\frac{\sum_{c=1}^{C}{\mathrm{TP}}_{c}}{N_{\mathrm{total}}}$$
  Here, *C* denotes the number of classes, and $N_{\mathrm{total}}=B\times H\times W$ is the total number of pixels, where *B* is the batch size and H×W are the spatial dimensions of the feature map. ${\mathrm{TP}}_{c}$ represents the number of true positives for class *c*. This formulation corresponds to the standard multi-class extension of OA, where the numerator accumulates correctly classified pixels across all classes and the denominator is the total pixel count. Unlike the binary formulation, which requires per-class true negatives (TN), this definition avoids ambiguity in multi-class evaluation and aligns with the actual protocol used in our experiments.
2. (mF1): Since high-resolution remote sensing images often suffer from class imbalance (e.g., large impervious surfaces versus small vehicle targets), relying solely on OA tends to cause biased evaluation. The F1 score, defined as the harmonic mean of Precision and Recall, provides a more rigorous assessment of model performance on challenging minority classes. It is computed as follows:
  (18) $$\left\{\begin{array}{c} \mathrm{Precision}=\frac{\mathrm{TP}}{\mathrm{TP}+\mathrm{FP}}\\ \mathrm{Recall}=\frac{\mathrm{TP}}{\mathrm{TP}+\mathrm{FN}}\\ F_{1}=2\times \frac{\mathrm{Precision}\times \mathrm{Recall}}{\mathrm{Precision}+\mathrm{Recall}} \end{array}\right.$$
  mF1 is then obtained by averaging the F1 scores over all classes.
3. (mIoU): mIoU is a widely adopted metric in dense prediction tasks. It not only accounts for classification correctness but also penalizes boundary over-segmentation (false positives) and under-segmentation (false negatives). It measures the ratio between the intersection and union of the ground-truth and predicted sets:
  (19) $$\mathrm{IoU}=\frac{\mathrm{TP}}{\mathrm{TP}+\mathrm{FP}+\mathrm{FN}}$$
  (20) $$\mathrm{mIoU}=\frac{1}{C}\sum_{i=1}^{C}{\mathrm{IoU}}_{i}$$
  where *C* is the total number of classes. In this paper, we place particular emphasis on the mIoU and F1 scores to validate the effectiveness of the frequency-spatial decoupling modules in mitigating semantic holes in areal objects and restoring rigid geometric boundaries.

### 4.3. Ablation Studies

To evaluate the individual contributions and collaborative effects of the three core modules—L-PPM, SA-WTFD, and EGA-Local—we conduct a structural ablation study on the Vaihingen and Potsdam datasets. The baseline model is constructed by removing all three modules from FSOD-Net, retaining only the ConvNext-Tiny backbone and the MDAF fusion module [38]. All subsequent ablation and efficiency comparisons are performed relative to this baseline. Starting from this configuration, we incrementally integrate each module and assess its performance impact. The analysis focuses on two primary metrics: mean Intersection over Union (mIoU) and mean F1-score (mF1).

#### 4.3.1. Effectiveness of EGA-Local

To verify the independent contribution of EGA-Local, we integrate it into the baseline model in isolation. Experimental results show that EGA-Local improves the mIoU to 83.90±0.10% and 86.64±0.07% on the Vaihingen and Potsdam datasets, respectively.

These performance gains highlight the importance of explicit modeling of microscopic rigid boundaries. As shown in Figure 6, the baseline’s continuous downsampling causes loss of high-frequency spatial details, leading to over-smoothing or structural breaks at the edges of buildings and roads. In contrast, EGA-Local embeds a fixed Laplacian operator, injecting explicit physical edge priors during feature extraction. Experimental results show that this physical constraint mitigates downsampling-induced blurring, preserves object contours in complex urban scenes, and improves discrimination of microstructures.

To further validate the rationale of the physical prior in EGA-Local, we individually integrate various edge operator variants into the baseline model and perform ablation experiments on both datasets. The quantitative comparison results are presented in Table 1.

The quantitative results suggest that explicit physical priors may offer advantages over purely implicit features in terms of local boundary modeling. As reported in Table 1, the baseline variant with conventional downsampling (i.e., w/Plain Conv) yields the lowest performance, with mIoU values of 83.55±0.07% and 86.1±0.06% on the Vaihingen and Potsdam datasets, respectively. This observation implies that, in the absence of explicit edge constraints, conventional convolutions may contribute to the loss of high-frequency boundary information of rigid objects during downsampling. When explicit physical edge operators—such as Sobel, Soft-Canny, and Laplacian—are incorporated, modest improvements in segmentation accuracy are observed. These findings are consistent with the hypothesis that embedding classical mathematical operators as structural priors can provide complementary guidance for boundary preservation.

Furthermore, the numerical differences also suggest varying degrees of scene adaptability among different operators. First-order gradient operators, such as w/Sobel and w/Soft-Canny, yield modest performance gains, though they may be less effective in handling complex feature distributions with diverse orientations and dense structures. In contrast, the Laplacian variant (w/Laplacian) employed in this study achieves mIoU values of 83.90±0.10% and 86.64±0.07% on the Vaihingen and Potsdam datasets, respectively. This may be attributed to the isotropic and zero-crossing properties of the Laplacian as a second-order differential operator, which enable it to capture intensity variations across multiple directions, thus potentially serving as a more flexible geometric constraint for boundary extraction in complex scenes.

To provide a more intuitive validation of the effectiveness of different physical edge priors within EGA-Local, Figure 7 illustrates the qualitative segmentation results of the baseline model upon the individual integration of each operator variant. By focusing on the complex object boundary regions highlighted by the red boxes in the figure, a progression in segmentation performance can be observed:

Observing the predictions of the first baseline variant (w/Plain Conv), networks relying exclusively on conventional convolutions for downsampling suffer from severe geometric structural degradation when processing dense remote sensing scenes. For instance, in the first and third rows, noticeable boundary blurring and inter-class feature aliasing occur at the interfaces between buildings and adjacent land covers (e.g., impervious surfaces or vegetation). This suggests that, in the absence of explicit physical constraints, conventional convolutions tend to incur the loss of high-frequency spatial details (i.e., rigid contours) during deep feature extraction. Following the integration of first-order differential operators (w/Sobel and w/Soft-Canny), the network’s edge awareness is notably enhanced, yielding moderate improvements in the sharpness of object contours. Nevertheless, man-made ground objects in remote sensing imagery frequently exhibit complex, multi-directional topological structures. Constrained by their inherent directional sensitivity, first-order operators struggle to accurately delineate non-orthogonal edges or complex corner points, thus failing to resolve the fragmentation of micro-structures (as highlighted by the red box in the fourth row).

In contrast, the second-order variant (w/Laplacian) adopted in this study exhibits detail fidelity and boundary-sharpening capabilities. As illustrated in the red-boxed regions, w/Laplacian not only separates closely adjacent buildings (first row) and reconstructs the rectilinear geometric boundaries of building rooftops (third row), but also captures and preserves micro-scale ground objects (fourth row). This high degree of visual consistency further validates the physical advantage of the second-order Laplacian operator’s isotropy. It is capable of sensitively capturing abrupt grayscale variations across all spatial directions, thereby retaining complete physical boundaries and providing the network with robust geometric guidance.

#### 4.3.2. Effectiveness of SA-WTFD

To verify the independent contribution of SA-WTFD in suppressing frequency-domain noise and purifying features, we integrate it into the baseline model in isolation. Experimental results show that SA-WTFD improves the mIoU to 83.85±0.09% and 86.51±0.06% on the Vaihingen and Potsdam datasets, respectively, as visually compared in Figure 8.

As illustrated in the visual comparisons, the complex textures of ground objects, high-frequency background clutter, and illumination and shadow effects are often highly coupled in the spatial domain of high-resolution remote sensing imagery. Owing to the lack of sensitivity to frequency-domain information, conventional baseline convolutional networks tend to misinterpret ineffective high-frequency noise as structural edges, inevitably leading to semantic confusion in complex regions. By incorporating the 2D Haar discrete wavelet transform, SA-WTFD achieves lossless feature decoupling in the frequency domain. More importantly, its subsequent spatial attention mechanism functions as a filter. The visualization results demonstrate that this module effectively suppresses irrelevant high-frequency background noise while preserving and enhancing geometric boundaries for segmentation tasks. This synergistic “frequency decoupling–spatial focusing” mechanism improves the purity of the network’s feature representations.

To further investigate the internal mechanisms and the necessity of individual components within SA-WTFD, we independently integrate various frequency-domain transformation variants of this module into the baseline model for quantitative ablation analysis on both datasets. The detailed experimental results are presented in Table 2.

Quantitative analysis indicates that, on the Vaihingen dataset, the variants employing the Fast Fourier Transform (w/FFT) and standard discrete wavelet transform (w/DWT) achieve mIoU values of 83.72±0.06% and 83.74±0.06%, respectively, across multiple independent runs. In comparison, the variant with SA-WTFD yields a slightly higher mIoU of 83.85±0.09%. A similar trend is observed on the Potsdam dataset, where SA-WTFD achieves 86.51±0.06%, marginally outperforming w/FFT (86.42±0.06%) and w/DWT (86.39±0.08%). In terms of mean F1-score, SA-WTFD achieves 91.11±0.09% and 92.59±0.07% on the two datasets, respectively. Although the absolute improvements are relatively modest, the consistent gains across multiple runs suggest that the integration of frequency-domain decomposition with spatial attention mechanisms within the SFFNet framework may offer a beneficial combination for feature representation.

To provide a more intuitive validation of the internal mechanisms governing SA-WTFD’s components, Figure 9 illustrates the qualitative segmentation results obtained by applying different variants to the baseline model. By focusing on the complex detail regions highlighted by the red boxes, a clear progression in segmentation quality driven by frequency-spatial collaborative processing can be observed:

First, the absence of local spatial information precipitates boundary distortions. As can be observed in the second column (w/FFT), the absence of time-frequency localization in the Fast Fourier Transform tends to produce less precise boundaries and some degree of confusion between buildings and adjacent land covers, as seen in the first and third rows. In contrast, when employing the discrete wavelet transform (w/DWT), object boundaries tend to appear jagged due to the interference of noise inherent in the raw high-frequency features.

Second, the spatial attention mask effectively prevents excessive smoothing of physical boundaries. A comparative analysis of the last two columns reveals that while global denoising without a mask suppresses internal noise, it tends to blunt the orthogonal edges and rigid contours of buildings (as highlighted in the red-boxed regions of the first and second rows). In contrast, the complete w/SA-WTFD variant exhibits detail fidelity. Driven by mask-guided adaptive selective filtering, this approach denoises homogeneous regions while preserving high-frequency physical boundaries. It not only achieves clear delineation of closely adjacent buildings but also retains micro-scale ground features (third row).

#### 4.3.3. Effectiveness of L-PPM

To evaluate the independent contribution of L-PPM to macro-context aggregation, we integrate it into the baseline model in isolation. Quantitative results show that L-PPM improves the mIoU to 83.79±0.07% and 86.55±0.06% on the two datasets, respectively.

As illustrated in Figure 10, where (a) is the baseline, (b) is the model with L-PPM, and GT denotes ground truth, the baseline model—constrained by the limited receptive field of local convolutions—struggles to establish long-range pixel dependencies when processing large-scale homogeneous planar features (e.g., expansive building rooftops). This often leads to semantic discontinuities and voids within contiguous regions. In contrast, Figure 10b demonstrates that, guided by the macro-contextual cues from L-PPM, the internal segmentation blind spots in the baseline are mitigated, and the overall semantic consistency of areal features is restored.

To evaluate the computational efficiency of L-PPM, we benchmark it against ASPP (DeepLab) and standard PPM (PSPNet)—two widely adopted multi-scale context aggregation methods in semantic segmentation.

As detailed in Table 3, the baseline model—without any context aggregation module—contains 0.38 M parameters, requires 3.06 G FLOPs, and achieves an inference speed of 800 FPS. Introducing ASPP and PPM raises the parameter count to 2.61 M and 1.63 M, respectively, increases FLOPs to 11.94 G and 7.91 G, and reduces inference speed to 383 FPS and 378 FPS. In contrast, L-PPM introduces only 0.64 M parameters and 4.14G FLOPs, while sustaining a rapid inference speed of 653 FPS. Compared to ASPP and PPM, L-PPM reduces the parameter count by 75.5% and 60.7%, lowers FLOPs by 65.3% and 47.6%, and accelerates inference by 70.3% and 72.7%, respectively. These results demonstrate that L-PPM achieves a balance between context modeling capability and computational cost, making it well-suited for resource-constrained deployment scenarios.

To further evaluate the computational efficiency of the proposed architecture, we compare the original SFFNet [38], a pure dual-stream baseline (Baseline), and our FSOD-Net under identical hardware conditions. Baseline is defined as the network stripped of SA-WTFD, L-PPM, and EGA-Local—retaining only Haar wavelet decomposition and standard convolutions—serving as the lower bound for efficiency assessment. SFFNet denotes the original frequency-spatial fusion network, while FSOD-Net represents our final improved design.

As presented in Table 4, Baseline achieves an inference speed of 48.90 FPS with a GPU memory footprint of 215.43 MB; however, its segmentation accuracy (mIoU) is 83.55±0.07%, reflecting the limited feature extraction capability of pure convolutional baselines in complex scenes. After incorporating the frequency-domain branch and attention mechanisms, the original SFFNet’s parameter count rises to 34.16 M, FLOPs reach 51.67 G, and inference speed drops to 42.99 FPS. In contrast, FSOD-Net removes global window attention and restructures the network with lightweight L-PPM, SA-WTFD, and EGA-Local. It contains 30.53 M parameters, reduces FLOPs to 36.87 G, while sustaining an inference speed of 47.65 FPS and a GPU memory footprint of 236.85 MB.

Quantitative comparisons show that FSOD-Net reduces parameters by 10.6%, lowers FLOPs by 28.6%, accelerates inference by 10.8%, and decreases GPU memory footprint by 8.4%, while preserving segmentation accuracy—validating the advantages of our module-level restructuring strategy in both accuracy and efficiency. Compared with Baseline, FSOD-Net increases parameter count and computation time by only 1.4% and 2.6%, respectively, while improving mIoU by 0.69%. These results indicate that the proposed method achieves a balance between segmentation accuracy and computational cost.

#### 4.3.4. Overall Efficiency and Synergistic Effects

To investigate the independent contributions and synergistic effects of the three core modules (L-PPM, SA-WTFD, and EGA-Local) in remote sensing semantic segmentation, we conduct structural ablation experiments on the ISPRS Vaihingen and Potsdam datasets. Using a baseline dual-stream network consisting solely of wavelet transform and standard convolutions as the reference, we progressively introduce each module for quantitative evaluation. The detailed ablation results are presented in Table 5.

As reported in Table 5, the baseline network achieves mIoU values of 83.55±0.07% and 86.15±0.06% on the Vaihingen and Potsdam datasets, respectively. Introducing each module individually into the baseline architecture leads to modest yet consistent improvements. Specifically, L-PPM alone yields mIoU of 83.79±0.07% and 86.55±0.06%, suggesting that lightweight pyramid pooling may facilitate the capture of macro-level context. Similarly, SA-WTFD (83.85±0.09% and 86.51±0.06%) and EGA-Local (83.90±0.10% and 86.64±0.07%) also show slight gains.

Beyond the independent contributions of each module, their combined configurations further reveal potential synergistic effects of the architectural restructuring. When L-PPM and SA-WTFD are jointly embedded, the model appears to benefit from complementary feature representations in macro-level semantic parsing and frequency-domain background clutter suppression. Meanwhile, the combination of L-PPM and EGA-Local tends to fill semantic gaps within large-scale objects while preserving local boundary rigidity, suggesting a cross-scale feature coordination. Finally, when all three modules are integrated, the full model achieves mIoU values of 84.24±0.11% and 86.79±0.09% on the Vaihingen and Potsdam datasets, respectively. The consistent improvements observed across multiple independent runs suggest that the primary advantage of this work may not lie in the individual contribution of any single component, but rather in the effective decoupling and recombination of frequency-domain denoising, macro-level pooling, and local physical priors within the SFFNet framework. This multi-dimensional synergy appears to contribute to mitigating feature conflicts in complex remote sensing tasks and may help enhance generalization robustness.

To verify the generalization capability and robustness of FSOD-Net across different feature extraction paradigms, we evaluate several mainstream backbones under identical experimental configurations. As presented in Table 6, the results confirm the key mechanisms underlying the proposed architectural design.

As presented in Table 6, when ConvNext-Tiny is adopted as the backbone—a modern pure convolutional architecture that incorporates macro-level design principles from Transformers—FSOD-Net achieves an mIoU of 84.24±0.11%, suggesting competitive architectural compatibility. In terms of computational efficiency, the model contains 30.53 M parameters and requires 36.87 G FLOPs, which are slightly higher than those of conventional residual networks, yet it maintains a reasonable inference speed of 47.65 FPS. These results indicate that the proposed frequency-spatial interaction mechanism may work effectively with backbones that offer larger receptive fields, providing a viable trade-off between segmentation accuracy and model complexity.

When using classical residual networks such as ResNet50 and ResNext50 as feature extractors, the model achieves mIoU values of 83.01±0.09% and 83.25±0.08%, respectively. Notably, the variant with ResNet50 attains 61.71 FPS with a low computational cost of 9.2G FLOPs. These results suggest that even with limited feature representation capacity and without relying on highly complex extractors, a reasonable reorganization of feature pooling and frequency-domain denoising mechanisms within the SFFNet framework may help maintain a relatively stable performance lower bound.

Finally, the efficiency comparison also points to a possible risk of structural redundancy when complex attention mechanisms are introduced into the backbone. ResNest50, which theoretically offers stronger representational capacity, achieves an mIoU of 83.59±0.09%, but at the cost of substantially higher computational load (42.41 G FLOPs) and lower inference speed (29.81 FPS). Its overall performance gain over the more compact ConvNext-Tiny is relatively limited. This may be partly attributed to feature overlap between the channel-wise recalibration in ResNest50 and the multi-scale aggregation strategy of FSOD-Net, which could lead to diminishing returns in the combined architecture.

Figure 11 illustrates the multi-dimensional feature response maps of FSOD-Net when processing complex remote sensing scenes. The visualizations reveal that the four decoupled branches exhibit a clear synergistic mechanism: the high-frequency branch sharpens and retains object boundaries via frequency-domain decoupling, with high-response regions clearly delineating the topological contours of buildings and roads. The low-frequency branch shows relatively high activation within homogeneous regions, potentially contributing to the suppression of interference from internal noise and clutter on semantic discrimination. In addition, global and local features are complementary at the receptive field level: global features ensure macro-level semantic continuity, while local features enable extraction of microscopic textures and fine-grained structures.

### 4.4. Comparative Experiments

To evaluate the proposed method in complex urban scenarios, we compare it with several mainstream high-resolution semantic segmentation networks on the ISPRS Vaihingen and Potsdam datasets. The selected comparison models cover a range of architectural paradigms, including: ABCNet [39], a convolutional neural network with spatial and contextual pathways; A2-FPN [40], which uses an attention-aggregated feature pyramid; BANet [41], a boundary-aware network; DC-Swin [42] and UNetFormer [43], both Transformer-based models with dense feature aggregation; SFFNet [38], a frequency-spatial fusion network; and SparseFormer [44], which introduces sparse feature representation.

To ensure a fair comparison, all comparison models are retrained on the same hardware platform (NVIDIA RTX 4500 GPU) using identical software and dataset splits. Since different network paradigms exhibit varying sensitivity to hyperparameters, we adopt the configurations recommended in their original papers. All models are trained for a fixed number of epochs as specified in Table 7, and the final-epoch weights are used for evaluation. The test set is not used for model selection or hyperparameter tuning, but solely for final performance reporting. Furthermore, the same test-time augmentation strategy is applied to all competing methods to ensure a consistent evaluation protocol.

Table 8 and Table 9 show the quantitative comparison of different semantic segmentation methods on the Vaihingen dataset.

Table 8 and Table 9 present, respectively, the per-class fine-grained accuracy and overall evaluation metrics of various mainstream semantic segmentation methods on the Vaihingen dataset. Over three independent runs, FSOD-Net achieves 91.35±0.09% in mean F1-score (mF1), 84.24±0.11% in mean IoU (mIoU), and 91.55±0.05% in overall accuracy (OA) on this dataset.

For small-scale object extraction and rigid boundary preservation, the “Car” category—being representative of such targets—is particularly susceptible to structural degradation during downsampling. As shown in Table 8, the proposed method achieves an F1 score of 90.85% on this category, showing a modest advantage over DC-Swin (88.90%) and SFFNet (90.34%). This improvement suggests that incorporating explicit physical priors, such as the Laplacian operator in the EGA-Local module, may help alleviate the smoothing effects of downsampling on object contours and serve as a structural complement for boundary preservation in dense scenes.

For the “Tree” and “ImSurf” categories, both characterized by irregular and complex textures, the model achieves F1 scores of 90.92% and 93.48%, respectively. These classes typically contain substantial high-frequency environmental noise and shadow interference, making them challenging for purely spatial-domain methods such as A2-FPN [40] and ABCNet [39]. In contrast, the proposed SA-WTFD mechanism, which combines frequency-domain decomposition with attention-based masking, helps suppress part of the irrelevant background noise during feature mapping. As a result, the model appears to show relatively improved robustness in handling areas with pronounced texture variations or shadow presence.

For large areal objects such as buildings and low vegetation, the proposed method achieves F1 scores of 95.83% and 85.66%, respectively. While some comparison models with dense self-attention or larger parameter counts obtain higher scores on individual categories—e.g., DC-Swin reaches 95.98% on buildings and ABCNet reaches 85.81% on low vegetation—our method still maintains a reasonably competitive performance level.

To further evaluate the segmentation performance in complex urban scenes, Figure 12 presents visual comparisons of our method against several representative models on the ISPRS Vaihingen dataset. The selected scenes cover diverse challenging scenarios, including densely clustered large buildings (rows 1–2), small objects partially occluded by shadows (row 3), and intricate boundary regions where vegetation and impervious surfaces are intermingled (row 5).

The qualitative results in Figure 12 suggest certain feature preferences and limitations among different network architectures when processing complex urban scenes. For large homogeneous regions such as the continuous building clusters in the first row, pure convolutional networks like ABCNet appear to struggle with global semantic consistency, often producing fragmented predictions with occasional internal voids. In contrast, Transformer-based models—including UNetFormer, DC-Swin, and SparseFormer—show improved coherence in macro-regions, likely due to their long-range dependency modeling or sparse aggregation mechanisms. However, these self-attention-based approaches may also lead to over-smoothing of high-frequency details during deep feature extraction, which could contribute to blurred contours and loss of fine structure in small objects, as seen in the vehicle cluster in the third row.

Second, when dealing with high-frequency textures and abrupt illumination changes, purely spatial learning methods still encounter difficulties in feature interpretation. Although A2-FPN introduces multi-scale feature aggregation and BANet incorporates boundary-aware mechanisms, some semantic ambiguity remains in areas with mixed shadows and vegetation, as shown in the fifth row. Meanwhile, SFFNet, as a baseline frequency-spatial fusion model, can enhance edge responses through wavelet transforms, but in the absence of explicit decoupling mechanisms, direct feature recombination may still introduce minor distortions in local spatial structure and some degree of classification redundancy.

In contrast, our method (Ours) shows relatively balanced adaptability across the aforementioned challenging scenarios. This visual improvement may be partly attributed to the proposed architecture, which decouples and reorganizes key feature extraction mechanisms during the feature mapping stage. On the one hand, the SA-WTFD module appears to help mitigate interference from complex textures and shadow noise, facilitating the recovery of structural details in shadowed regions, such as the vehicle in the third row. On the other hand, the global context extracted by L-PPM and the local edge priors provided by EGA-Local seem to complement each other during feature fusion, contributing to better semantic coherence in large-scale objects (rows 1–2) and improved boundary delineation in small objects and transition zones (row 5).

To further evaluate the optimization efficiency and convergence behavior of the proposed network during training, Figure 13 presents locally zoomed-in curves of the training-set mIoU as a function of training epoch. These curves compare the proposed FSOD-Net with five comparison models on the Vaihingen dataset over the first 45 epochs. All comparison models were trained to completion under their respective maximum epoch limits listed in Table 7. The first 45 epochs were selected as a common observation window to visualize the initial convergence dynamics and the local effect of the first learning-rate hot restart on optimization.

During the initial training phase (Epochs 0–15), FSOD-Net exhibits relatively fast convergence. Compared with other comparison models, it achieves a higher starting accuracy and a steeper performance growth curve, reaching an mIoU of 80% within a shorter period. This suggests that the frequency-spatial decoupling architecture may help reduce feature coupling losses in the early stages of training.

With the cosine annealing strategy with warm restarts, the learning rate undergoes its first reset at Epoch 15. To illustrate the local effects of this mechanism, Figure 13 shows the training curves for the first 45 epochs, which cover the first two annealing cycles of 15 and 30 epochs. Around the reset points, most compared models show some degree of fluctuation and performance drop, particularly the purely spatial networks such as ABCNet and BANet. In contrast, FSOD-Net exhibits relatively smaller oscillations and recovers more quickly after the learning rate stabilizes.

To further evaluate the generalization capability and robustness of our method in higher-resolution and more complex urban scenes, we conduct comparative experiments on the ISPRS Potsdam dataset. Compared with Vaihingen, Potsdam features denser building clusters, more intricate shadow patterns, and more heterogeneous land-cover distributions, imposing stricter requirements on fine-grained feature extraction and long-range semantic reasoning.

Table 10 and Table 11 summarize the per-class accuracy and overall metrics of various methods on the ISPRS Potsdam dataset. Compared with Vaihingen, Potsdam offers higher spatial resolution, more complex structures, and richer textures, which pose greater challenges for segmentation. Over three independent runs, FSOD-Net achieves 92.81±0.07% in mF1, 86.79±0.09% in mIoU, and 91.45±0.05% in OA.

In terms of overall performance, the proposed method shows a modest improvement in average mIoU compared with the baseline SFFNet and the representative DC-Swin model. The observed gain suggests that the SA-WTFD frequency-domain denoising mechanism, when combined with the multi-scale aggregation of L-PPM, may contribute to feature refinement and semantic retention during the feature mapping stage, particularly given the complex textures and high-frequency noise present in the Potsdam dataset.

For the building category, our method achieves an F1 score of 96.05%, roughly comparable to DC-Swin (96.07%). For low vegetation, it reaches 87.66%. These results suggest that, even without global self-attention modules, the network can still achieve reasonably competitive performance on large-area objects by effectively combining spatial pooling and frequency-domain feature processing.

For challenging categories such as cars, impervious surfaces, and trees, the proposed method achieves F1 scores of 96.78%, 93.87%, and 89.69%, respectively, showing modest improvements over the baseline. In complex urban scenes, where edges of low vegetation and vehicles often intertwine with building shadows, feature aliasing is a common issue in deep convolutional networks. The integration of explicit physical priors, such as the Laplacian operator in EGA-Local, appears to help alleviate the smoothing effect of downsampling on small object contours. These results support the view that the improved balance between local detail and global semantics in our model is not solely due to any single component, but rather stems from the architectural reorganization and combination of macro-contextual features, edge priors, and frequency-domain denoising within the SFFNet framework.

To evaluate the visual segmentation performance of our method in complex urban scenes, we compare it against seven comparison models on the ISPRS Potsdam dataset. As illustrated in Figure 14, the selected regions (highlighted by red boxes) include small-scale targets, shadow-occluded objects, and ambiguous semantic boundaries.

The accurate extraction of small targets or weakly characterized objects in high-resolution remote sensing imagery is often hindered by spatial information loss caused by downsampling in deep networks. As shown in the first and second rows of Figure 14, when scenes contain densely distributed small-scale targets with complex background interference, most comparison models exhibit varying degrees of difficulty in feature discrimination. Specifically, in the red-boxed region at the bottom of the second row, several purely spatial models struggle to identify small rectangular objects, often confusing them with surrounding impervious surfaces. In the fifth row, Transformer-based models such as UNetFormer and SparseFormer, despite their strong global perception, still show some boundary sticking along building outlines. In contrast, our method delineates the geometric transition between buildings and impervious surfaces more clearly in the fifth row, and also recovers some easily missed small targets in the second row. This visual improvement suggests that the decoupled frequency-domain denoising and spatial feature extraction may help alleviate the loss of high-frequency local details, offering potentially better robustness in noise-prone scenarios.

Furthermore, transition zones between land covers with complex textural variations and similar spectral characteristics tend to cause spatial semantic ambiguity in model predictions. As shown in the third row of Figure 14, subtle background noise appears along the ambiguous boundary between trees and impervious surfaces in the red-boxed region. Some comparison models show blurred edge structures or localized misclassifications in this area, while certain attention-based models even produce fragmented and isolated patches. In contrast, our method generates masks that maintain better spatial consistency with the ground truth, suggesting that the proposed approach helps alleviate semantic confusion caused by feature overlap.

## 5. Conclusions

To address the challenges of high-frequency noise interference and ambiguous boundaries in high-resolution remote sensing semantic segmentation, we propose FSOD-Net, a Frequency-Spatial Feature Decoupling Network built upon the SFFNet framework. Unlike conventional approaches that rely heavily on complex attention mechanisms, the main contribution of this work lies in the effective decoupling and reorganization of frequency-domain transforms, spatial pooling, and physical edge priors within the network architecture, which helps alleviate feature coupling conflicts in complex remote sensing scenarios.

The core findings and reconstruction advantages of this study are summarized below.

First, in the feature local constraint stage, the proposed method improves upon the conventional implicit feature learning paradigm. Ablation results suggest that embedding a second-order Laplacian operator—characterized by its isotropic and zero-crossing properties—as an explicit geometric prior (EGA-Local) helps alleviate the smoothing effect of standard convolutions on microscopic rigid boundaries during downsampling. This provides a complementary mechanism for mitigating inter-class adhesion in dense scenes.

Second, in frequency feature processing, the proposed approach combines discrete wavelet transform (DWT) with a Spatial Attention (SA) mask (SA-WTFD). This design circumvents the inherent limitation of the Fast Fourier Transform (FFT)—namely, the lack of spatiotemporal localization—and provides the network with adaptive filtering capability. As a result, the model can suppress high-frequency environmental noise while preserving boundary-related high-frequency responses that are beneficial for segmentation.

Finally, we validate the generalization potential of the proposed reconstruction mechanism on modern pure convolutional architectures. Experimental results on the ISPRS Vaihingen and Potsdam datasets show that FSOD-Net with ConvNext-Tiny as the backbone exhibits competitive architectural adaptability. This supports the view that, without relying on complex internal attention mechanisms, a reasonable trade-off between local detail representation and global semantic coherence can be achieved through the decoupling and recombination of frequency and spatial feature dimensions.

In summary, FSOD-Net offers an alternative solution for fine-grained remote sensing segmentation, grounded in physical prior guidance and frequency-spatial joint denoising. It also demonstrates the potential of integrating classical signal processing principles into deep neural networks. Future work will focus on lightweight architecture design for real-time inference on resource-constrained satellite or UAV edge devices. We also plan to extend the collaborative mechanism to multimodal feature fusion tasks, targeting more complex all-weather Earth observation scenarios.

## Figures and Tables

**Figure 1:**
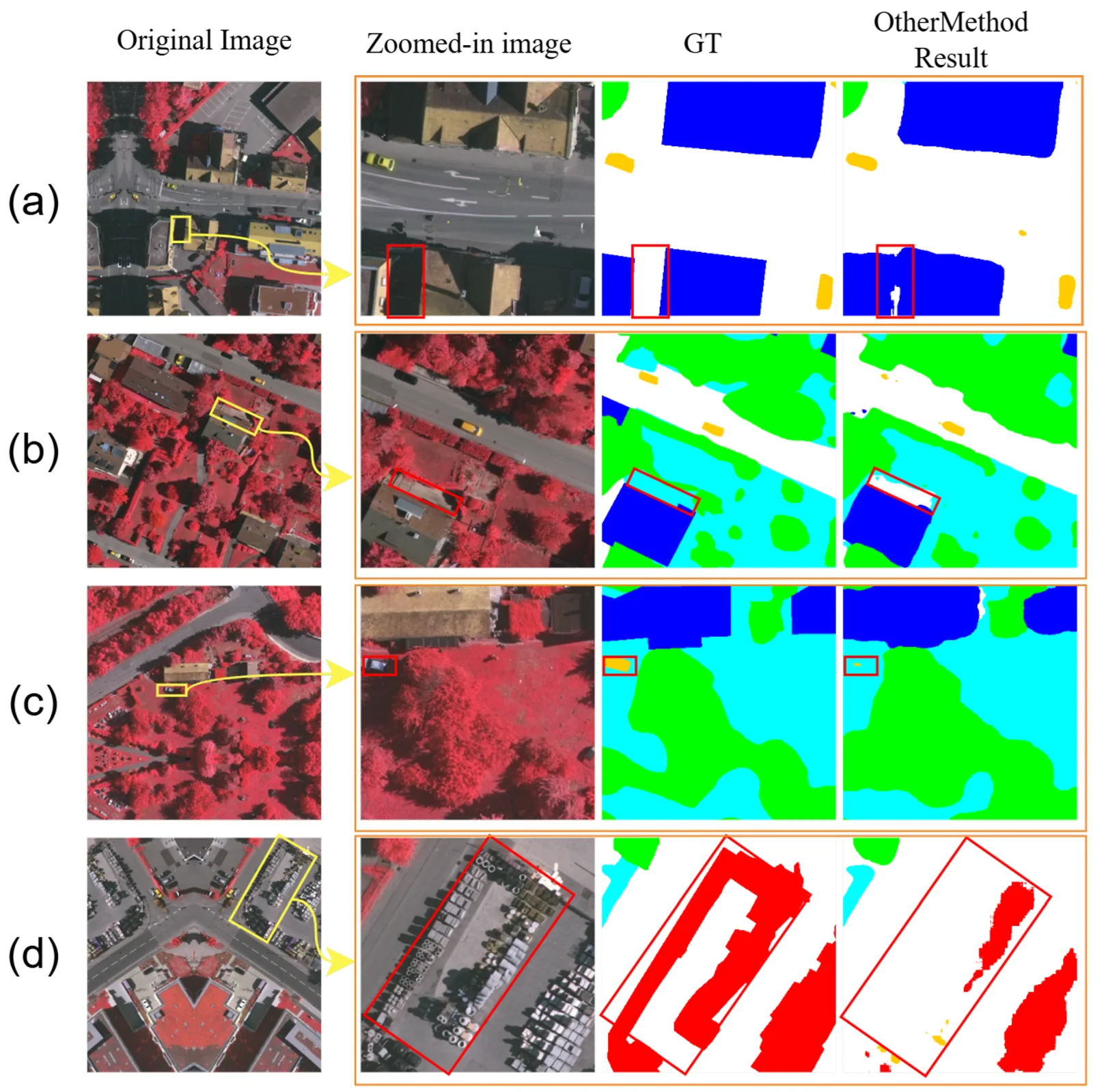
The challenges of semantic fragmentation and boundary loss in complex remote sensing scenarios. The figure illustrates, from left to right: the original image, a locally enlarged view, the ground truth (GT), and the prediction results generated by the BANet model. Specifically, panels (**a**,**d**) demonstrate scenarios with large homogeneous areas and complex shadows. Constrained by limited local receptive fields and direct feature coupling, spatial models lacking effective guidance from global context and high-frequency information are prone to semantic fragmentation and internal voids. Panels (**b**,**c**) represent transition regions between micro-boundaries and small-scale targets. In these areas, the excessive feature smoothing induced by deep network downsampling prevents spatial-domain-centric models from accurately delineating the fine physical contours of objects.

**Figure 2:**
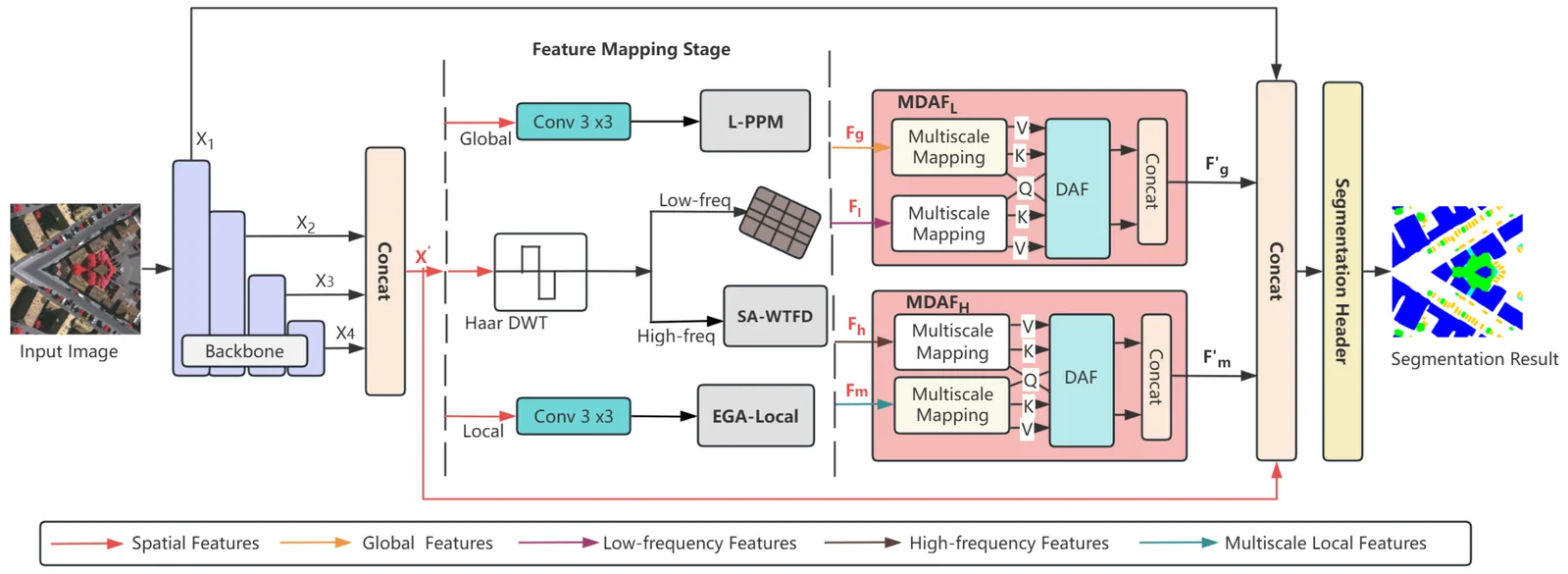
Overall architecture diagram.

**Figure 3:**
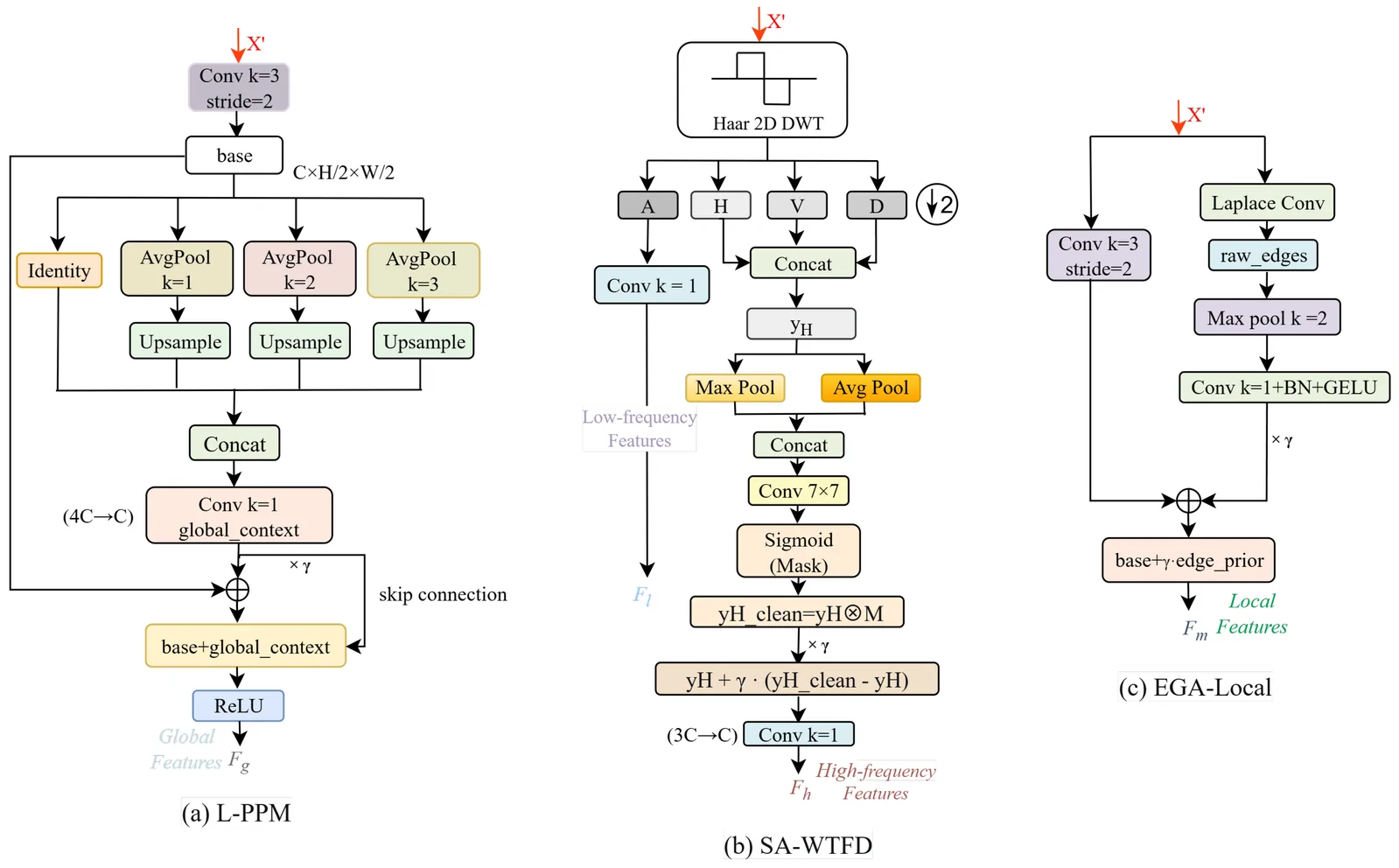
Diagram of core modules. This figure illustrates the three decoupled feature mapping branches in the feature mapping stage. (**a**) L-PPM aggregates macro-contextual semantics through parallel multi-scale adaptive average pooling. (**b**) SA-WTFD decomposes the features via the Haar wavelet transform and applies an adaptive spatial attention mask to suppress high-frequency background clutter while preserving true geometric edges. (**c**) EGA-Local embeds a fixed Laplacian operator as an explicit physical edge prior to preserve micro-scale rigid boundaries during downsampling.

**Figure 4:**
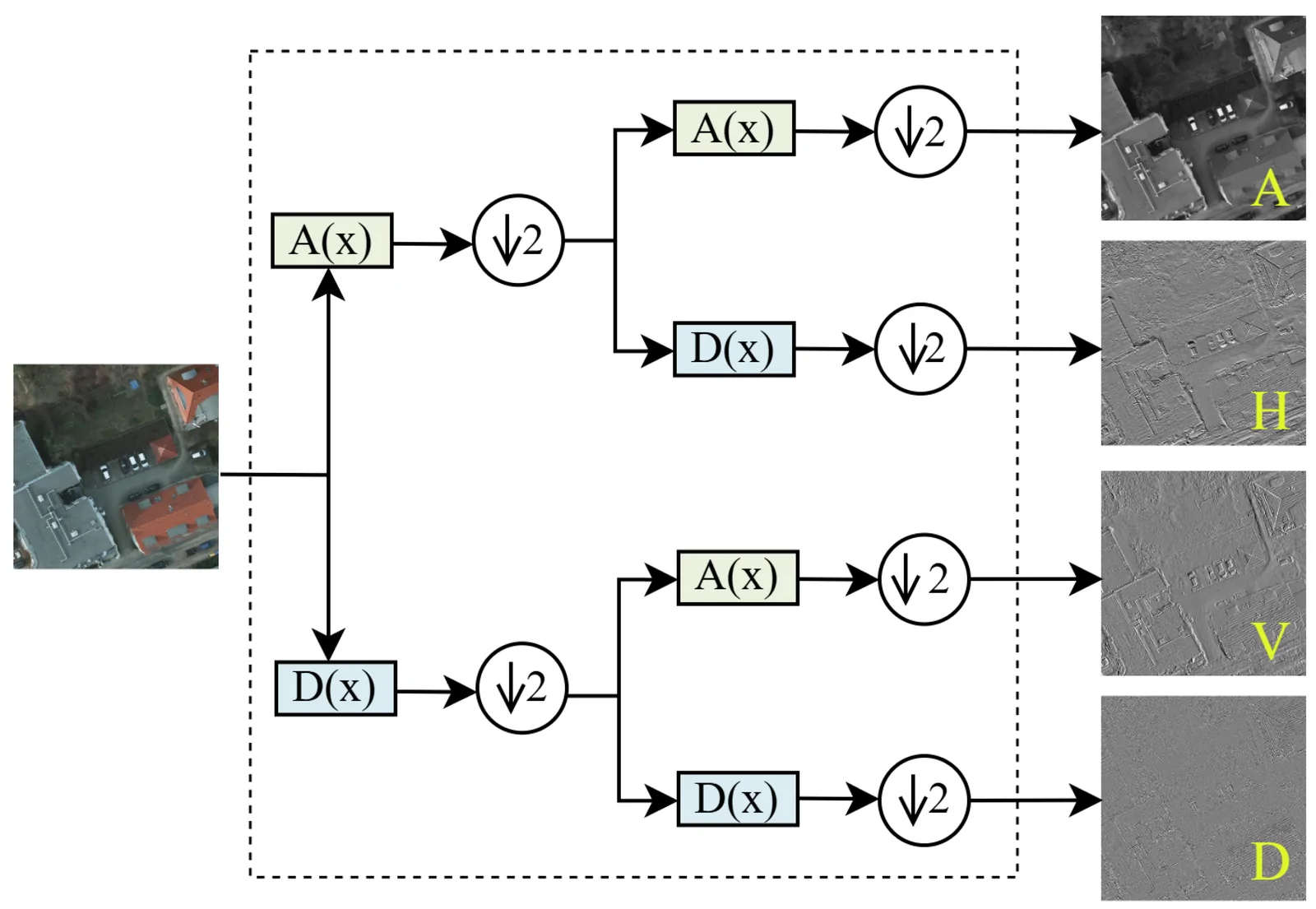
Schematic diagram of the 2D Haar discrete wavelet transform decomposition. *A* denotes the low-frequency approximation coefficients obtained via low-pass filtering, while *H*, *V*, and *D* denote the horizontal, vertical, and diagonal high-frequency detail coefficients obtained via high-pass filtering, respectively. Each decomposition step halves the spatial resolution of the feature map through downsampling (indicated by the down arrows).

**Figure 5:**
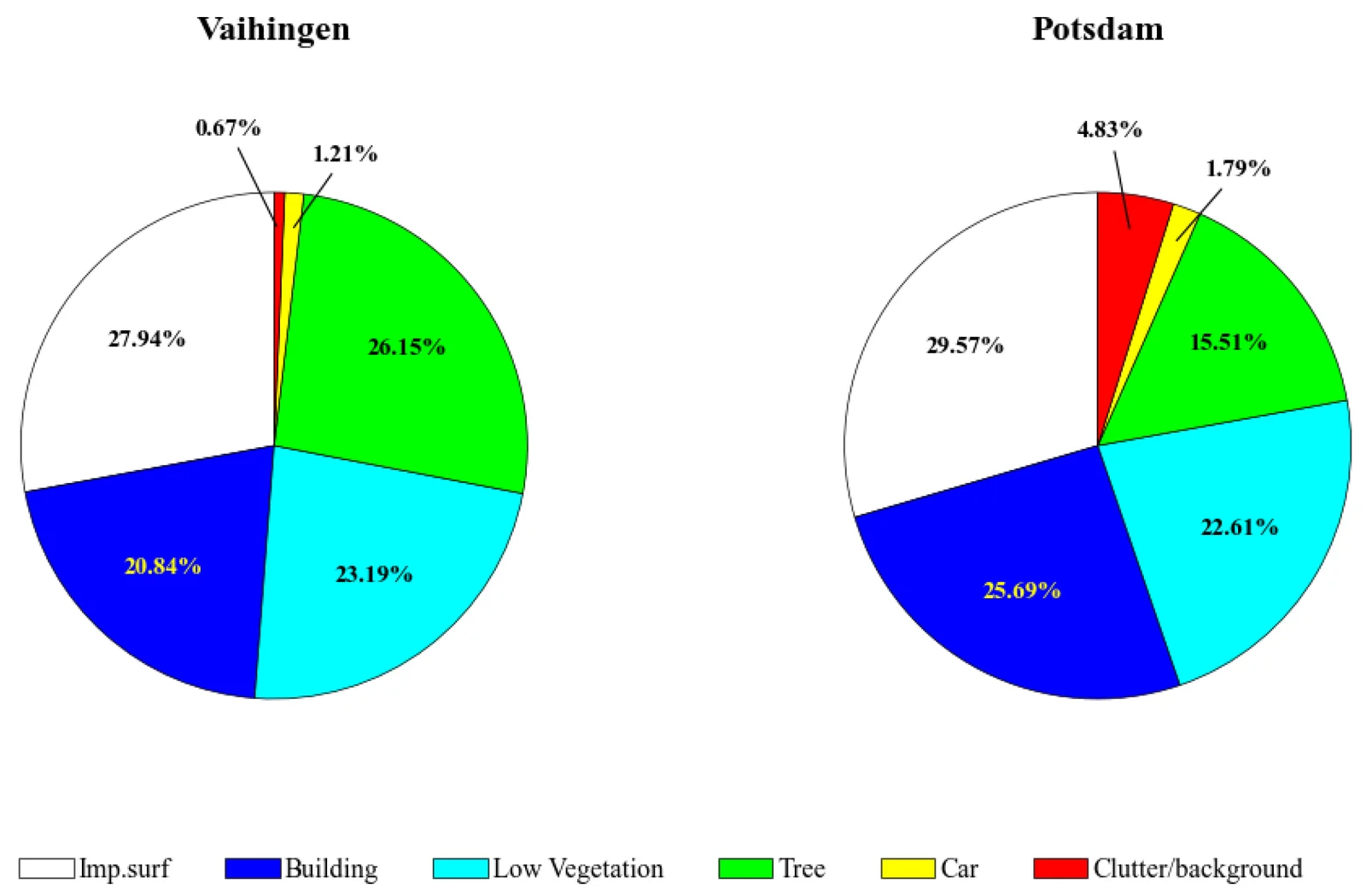
Class distribution of the Vaihingen and Potsdam datasets.

**Figure 6:**
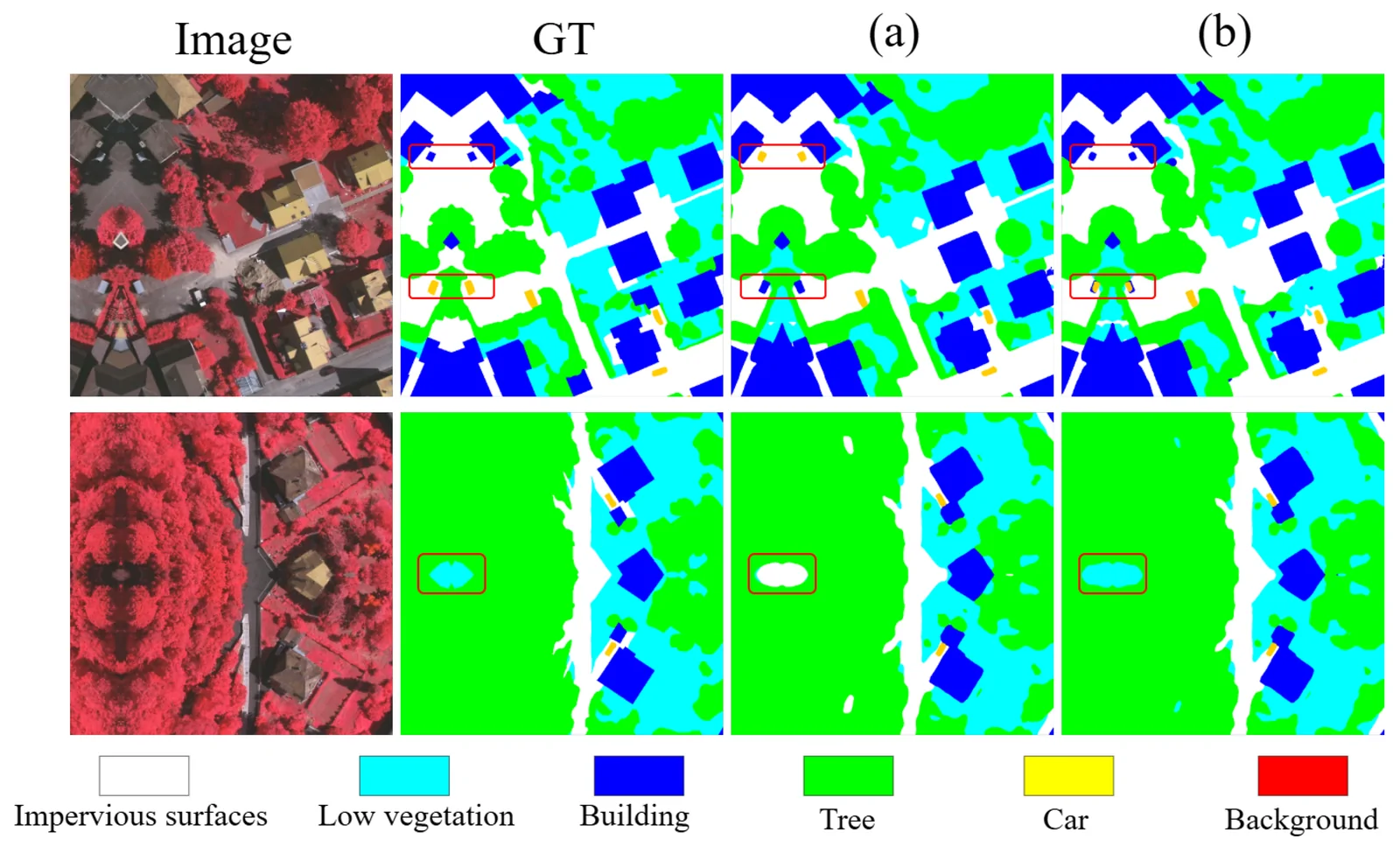
Results of the EGA-Local module. (**a**) Segmentation results of the baseline model. (**b**) Segmentation results of the baseline model augmented with the EGA-Local module. GT denotes the ground truth.

**Figure 7:**
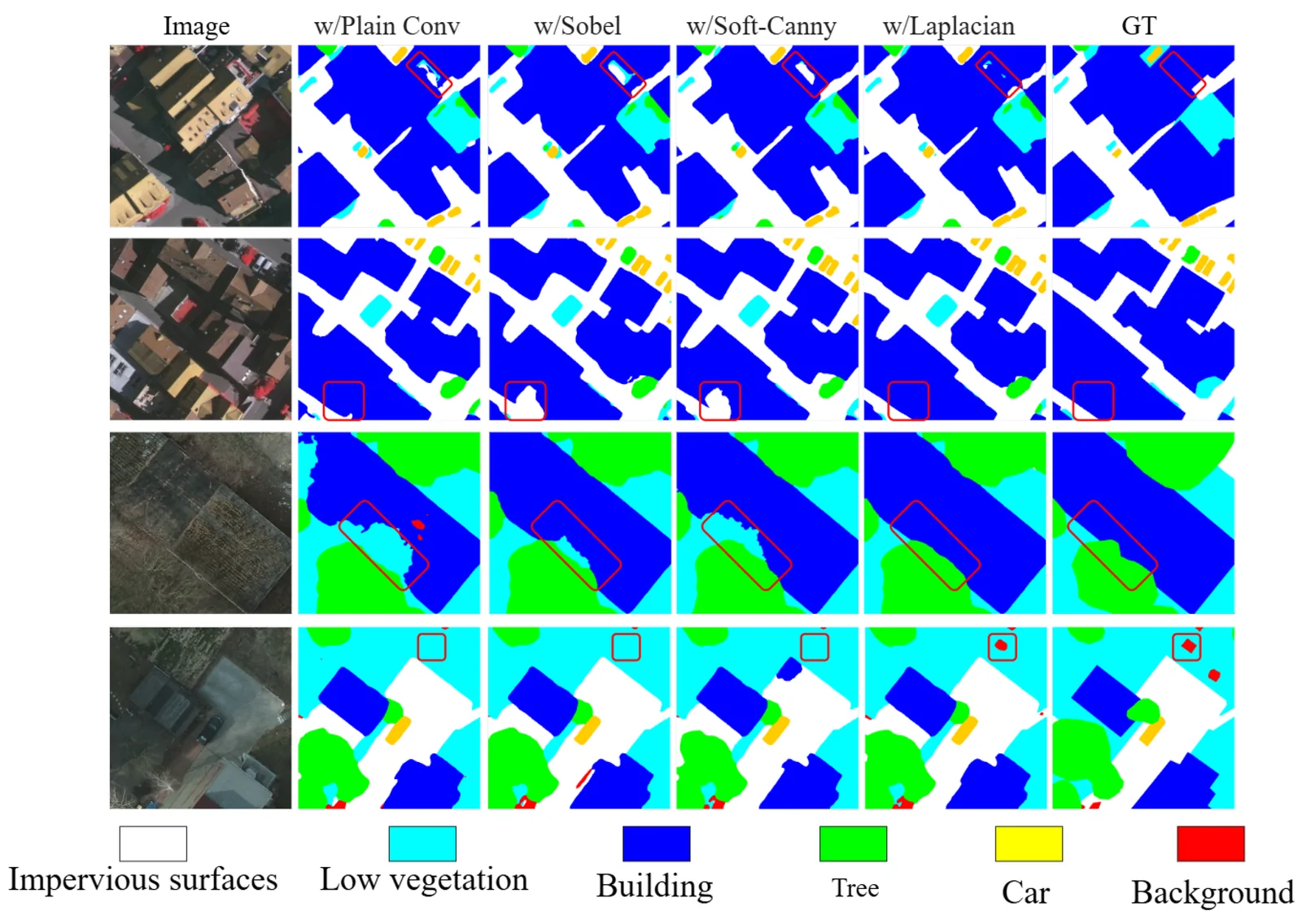
Qualitative visual comparison of different edge prior variants within the EGA-Local module. From left to right, the columns display the original input Image, segmentation results using plain convolution (w/Plain Conv), the Sobel operator (w/Sobel), the Soft-Canny operator (w/Soft-Canny), the Laplacian operator (w/Laplacian), and the Ground Truth (GT). The red boxes highlight specific local regions to illustrate the differences in preserving micro-scale boundaries among various operators.

**Figure 8:**
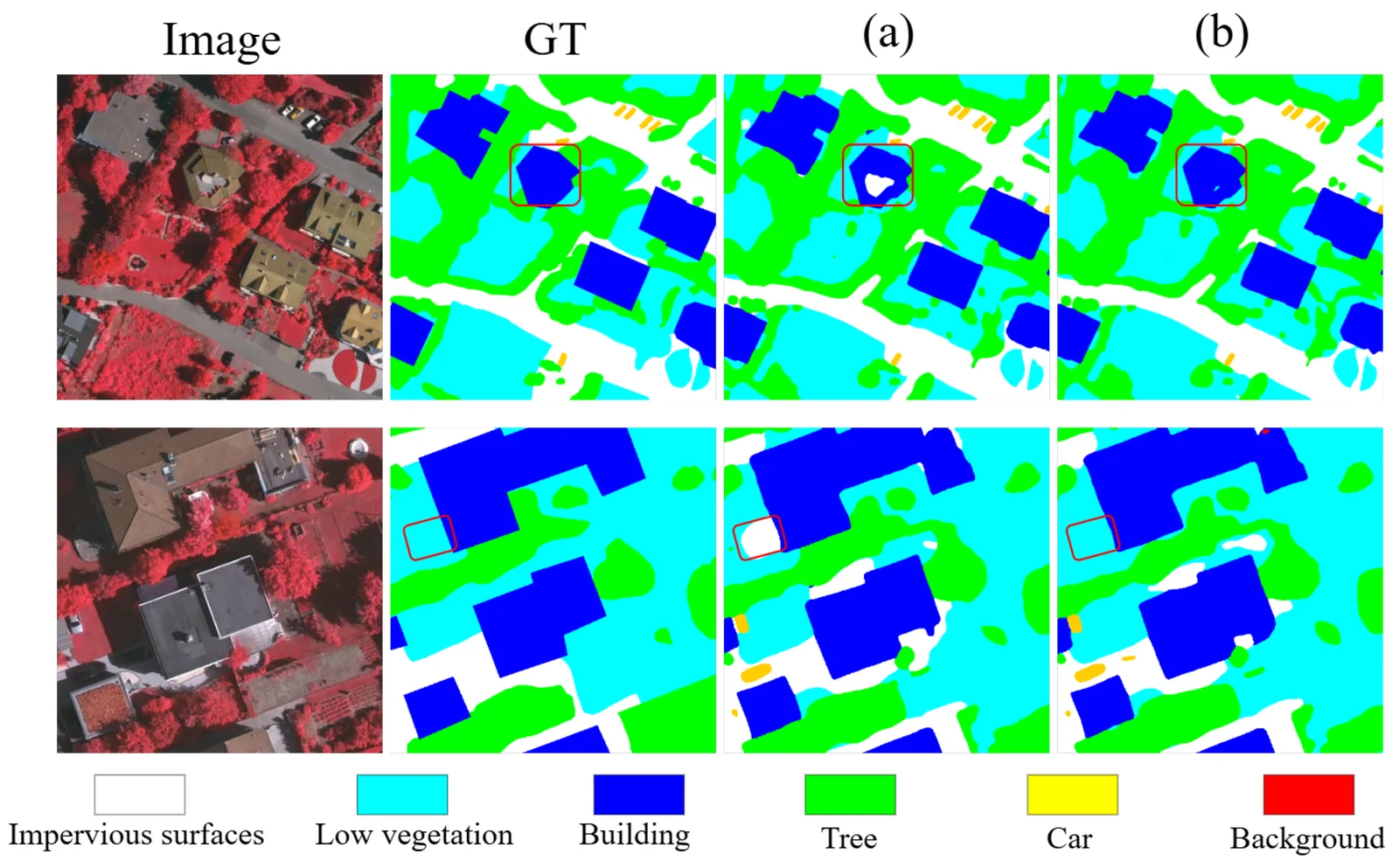
Performance of the SA-WTFD module. (**a**) Segmentation results of the baseline model. (**b**) Segmentation results of the baseline model augmented with the SA-WTFD module. GT denotes the ground truth. The red frames highlight specific local regions to illustrate the effectiveness of the SA-WTFD module in suppressing high-frequency background noise and preserving structural edges.

**Figure 9:**
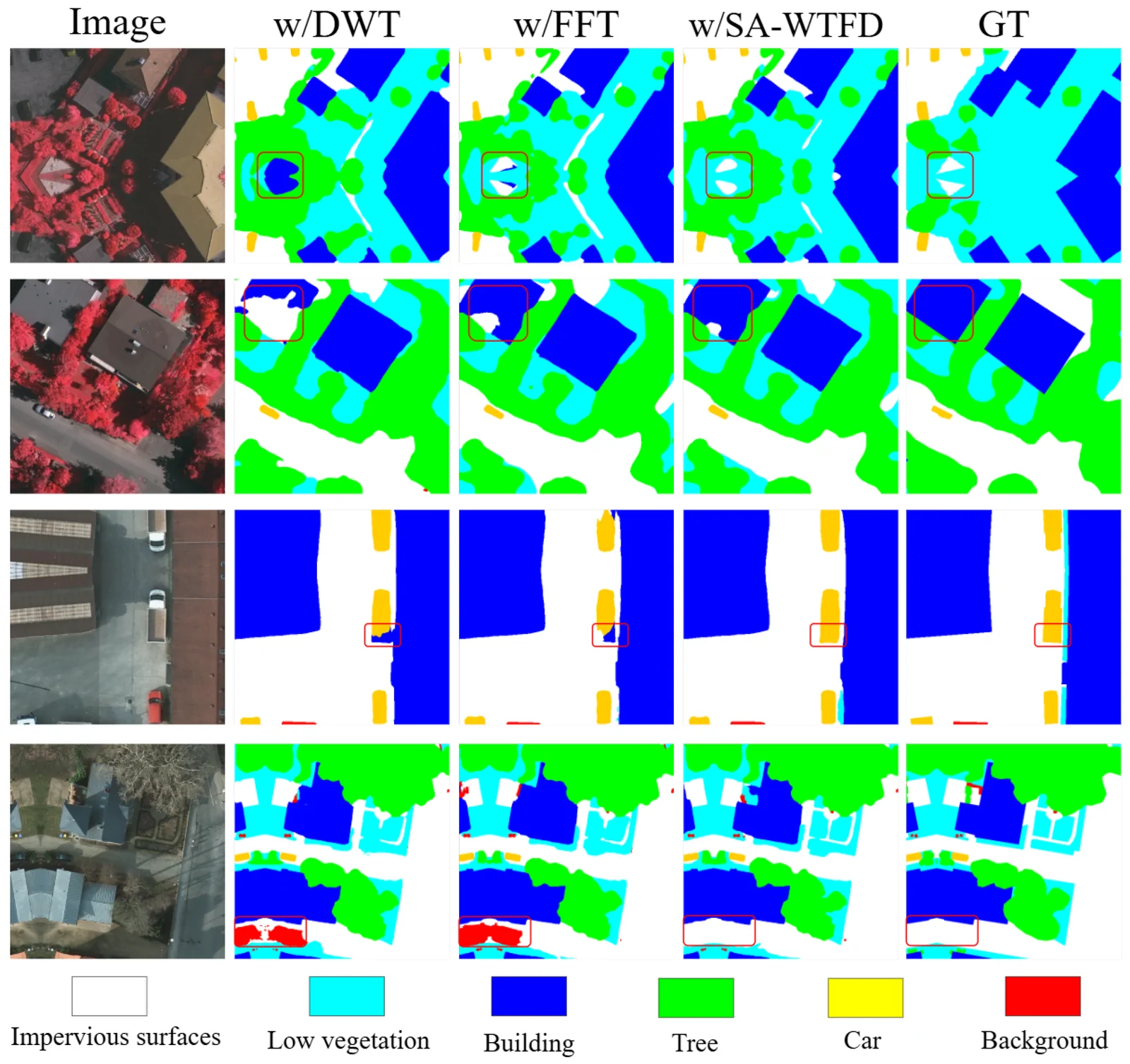
Qualitative visual comparison of different variants within the SA-WTFD module. From left to right, the columns display the original input Image, segmentation results using standard Discrete Wavelet Transform (w/DWT), Fast Fourier Transform (w/FFT), the proposed Spatial Attention Wavelet Transform Feature Decomposer (w/SA-WTFD), and the Ground Truth (GT). The red frames highlight specific local regions to illustrate the effectiveness of different variants in suppressing high-frequency background noise and preserving structural edges.

**Figure 10:**
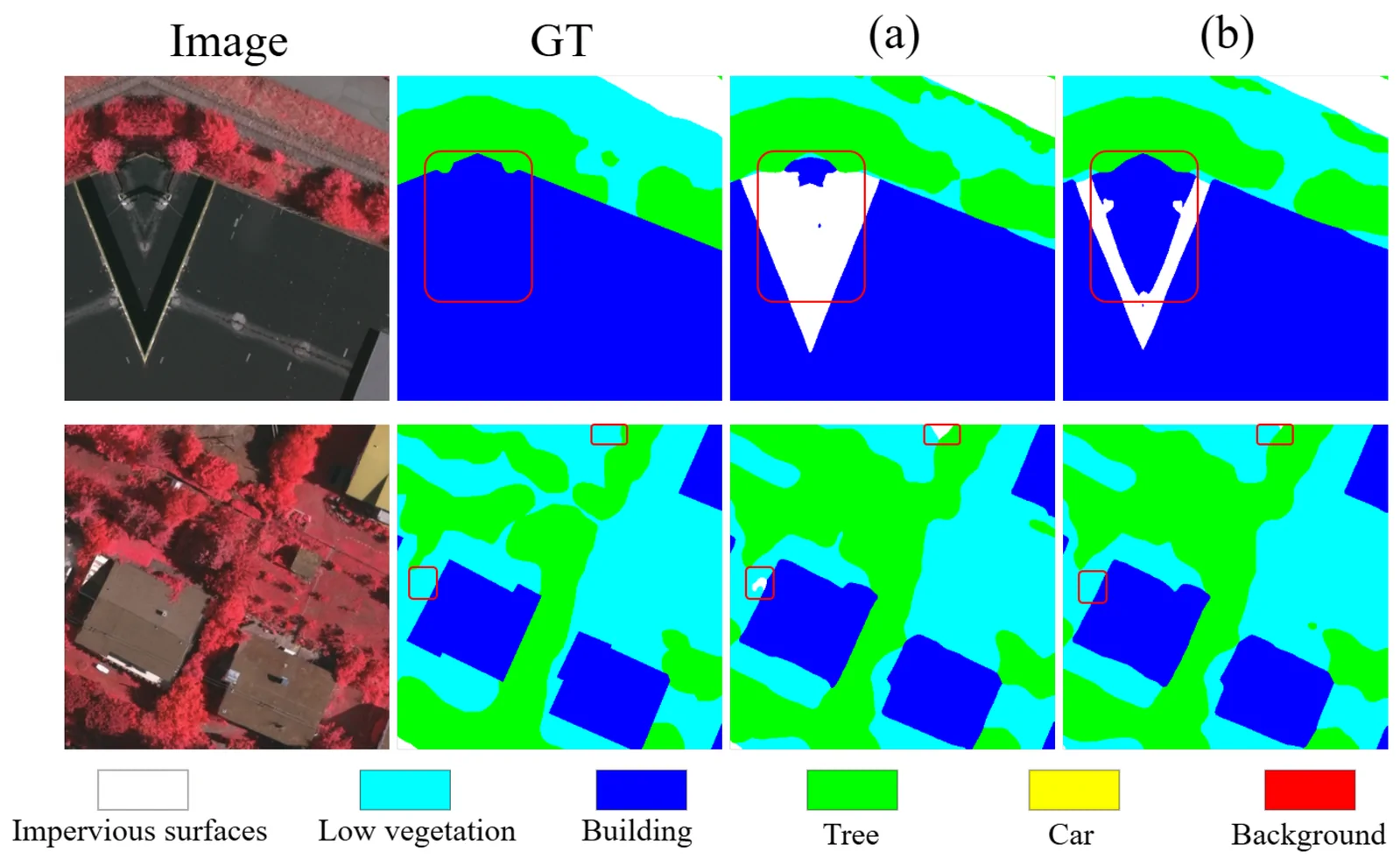
Performance of the L-PPM module. (**a**) Segmentation results of the baseline model. (**b**) Segmentation results of the baseline model augmented with the L-PPM module. GT denotes the ground truth.

**Figure 11:**
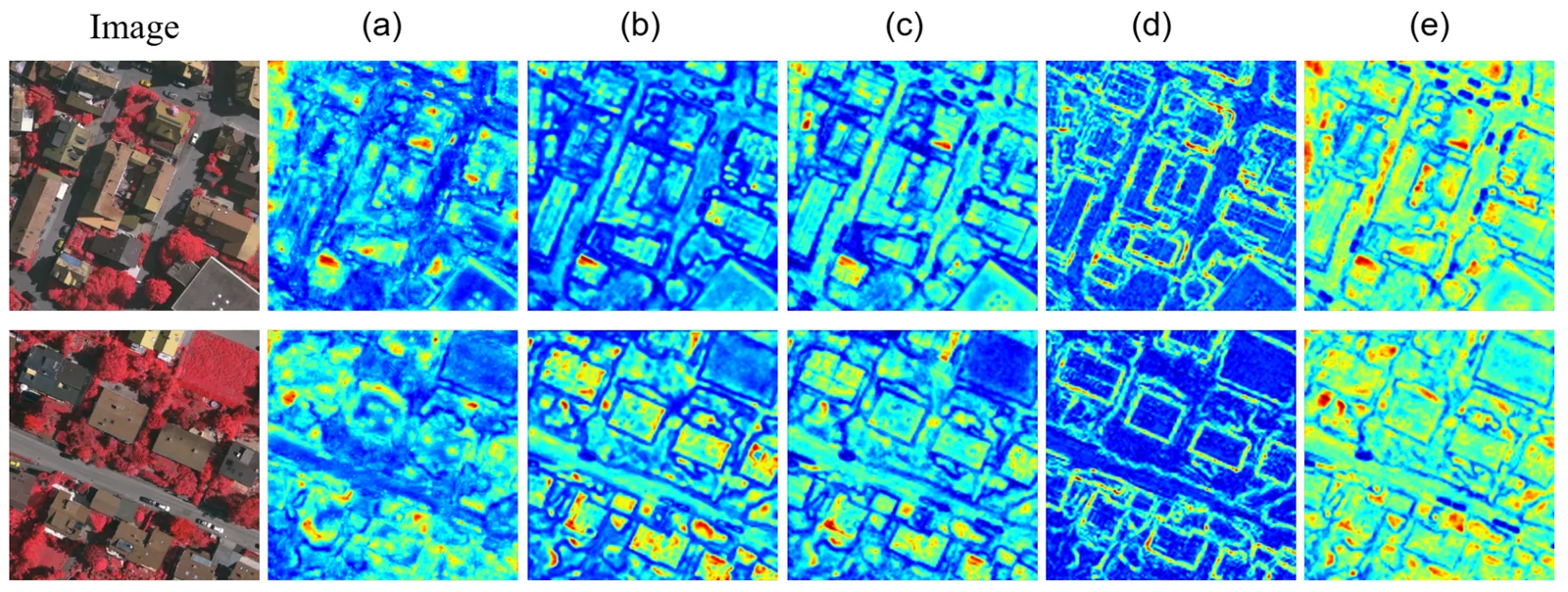
Visualization of multidimensional feature responses. (**a**) Raw features. (**b**) Global features. (**c**) Local features. (**d**) High-frequency features. (**e**) Low-frequency features.

**Figure 12:**
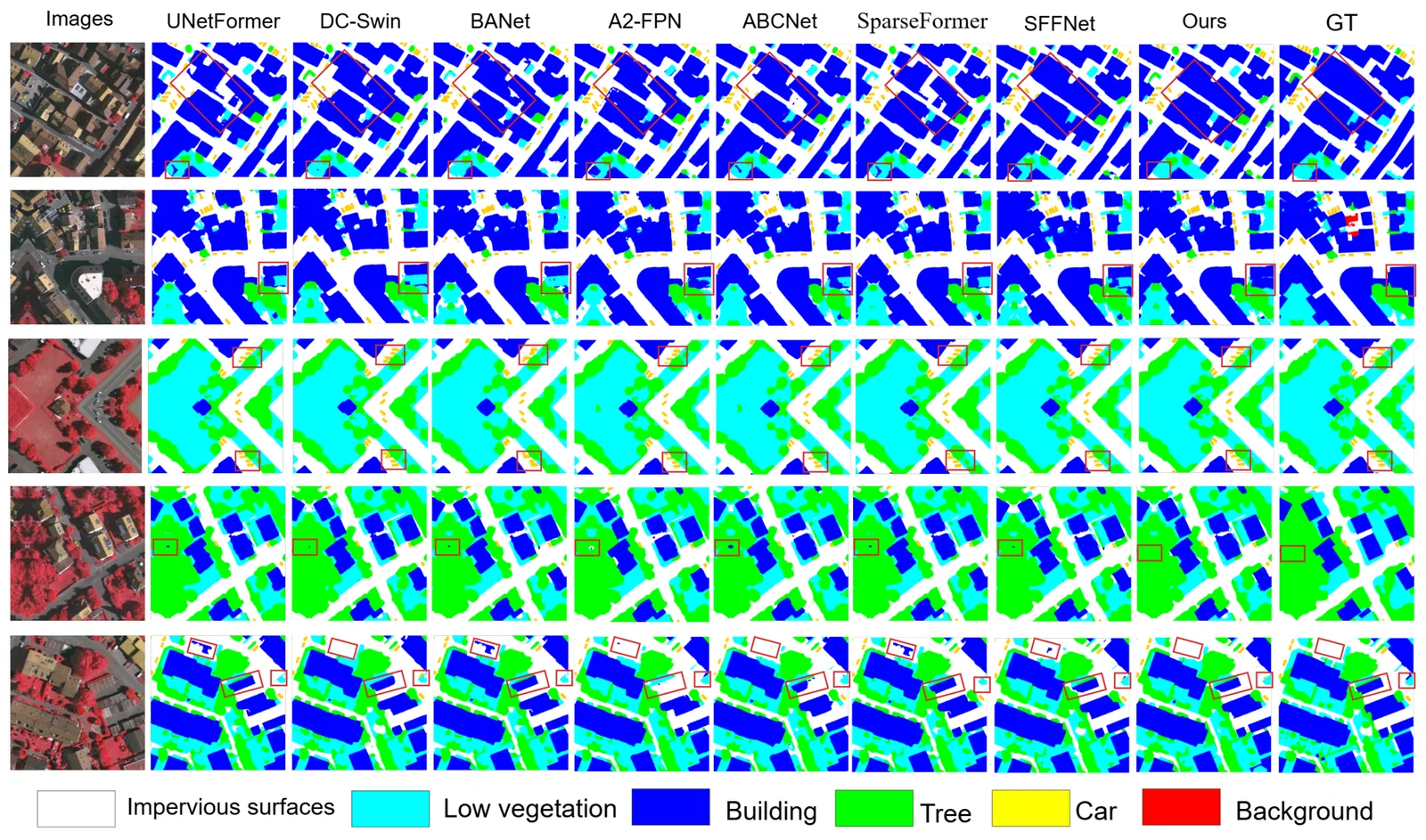
Visualizations of the various models on the Vaihingen dataset. The columns from left to right correspond to: original Images, UNetFormer [43], DC-Swin [42], BANet [41], A2-FPN [40], ABCNet [39], SparseFormer [44], SFFNet [38], our model (Ours), and Ground Truth (GT). The red frames highlight specific local regions for detailed visual comparison. From the figure, it can be observed that our model performs better in preserving precise micro-scale boundaries and intricate object contours compared to other spatial segmentation methods, while also effectively suppressing high-frequency background clutter.

**Figure 13:**
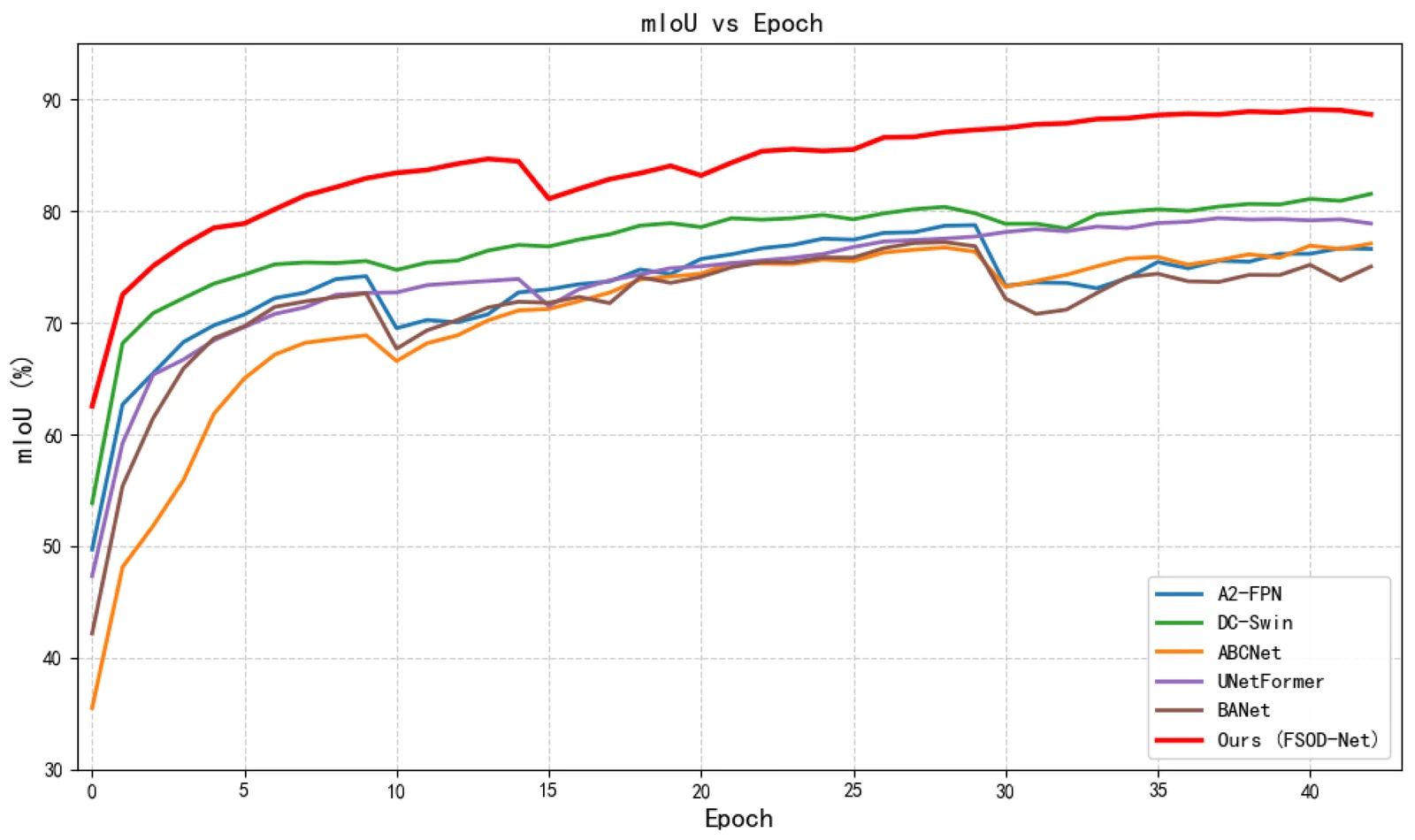
Curves of mIoU versus training epochs. (Note: To better illustrate the transient influence of the learning rate warm-restart mechanism on the optimization dynamics, this figure presents a magnified view of the convergence curves for the first 45 epochs).

**Figure 14:**
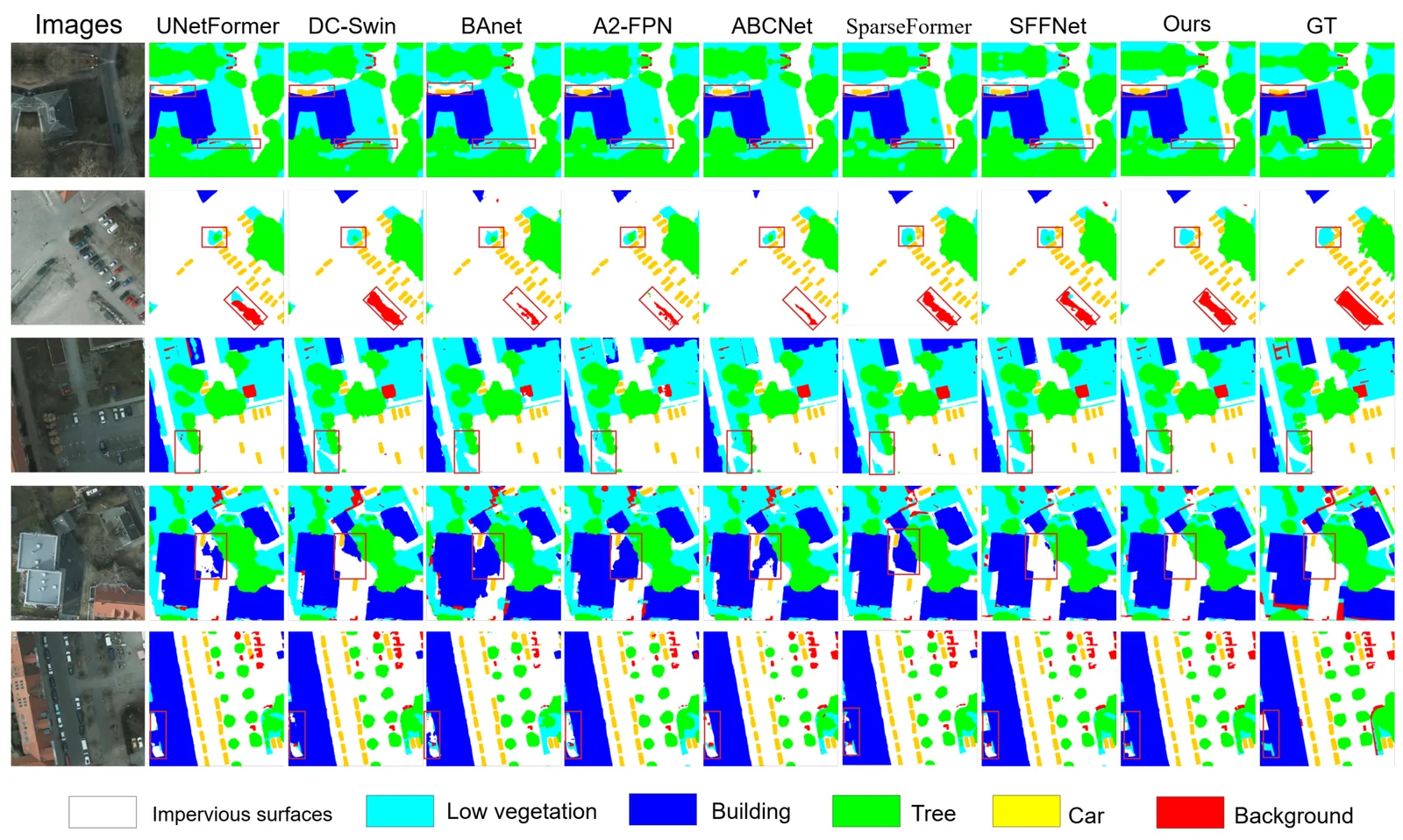
Visualizations of the various models on the Potsdam dataset. The columns from left to right correspond to: original Images, UNetFormer [43], DC-Swin [42], BANet [41], A2-FPN [40], ABCNet [39], SparseFormer [44], SFFNet [38], our model (Ours), and Ground Truth (GT). The red frames highlight specific local regions for detailed visual comparison.

**Table 1:** Results of quantitative ablation experiments for different edge prior operators in the EGA-Local module.

Method	Vaihingen		Potsdam	
	F1 (%)	mIoU (%)	F1 (%)	mIoU (%)
w/Plain Conv	90.68±0.09	83.55±0.07	92.36±0.11	86.15±0.06
w/Sobel	90.93±0.07	83.57±0.06	92.57±0.08	86.41±0.06
w/Soft-Canny	91.05±0.08	83.81±0.07	92.66±0.06	86.59±0.09
w/Laplacian	91.12±0.09	83.90±0.10	92.72±0.06	86.64±0.07

**Table 2:** Results of ablation comparison experiments for different frequency-domain transformation methods in the SA-WTFD module.

Method	Vaihingen		Potsdam	
	F1(%)	mIoU (%)	F1 (%)	mIoU (%)
w/FFT	90.99±0.08	83.72±0.06	92.48±0.07	86.42±0.06
w/DWT	91.02±0.07	83.74±0.06	92.47±0.06	86.39±0.08
w/SA-WTFD	91.11±0.09	83.85±0.09	92.59±0.07	86.51±0.06

**Table 3:** Comparison of the efficiency of different context aggregation modules.

Module	Params(M) ↓	FLOPs(G) ↓	FPS ↑
Baseline	0.38	3.06	800
w/ASPP	2.61	11.94	383
w/PPM	1.63	7.91	378
w/L-PPM	0.64	4.14	653

**Note:** ↓ indicates that a lower value is better, while ↑ indicates that a higher value is better.

**Table 4:** Efficiency of network architectures.

Method	Params (M) ↓	FLOPs (G) ↓	FPS ↑	VRAM (MB) ↓
Baseline	30.13	35.15	48.90	215.43
SFFNet	34.16	51.67	42.99	258.45
FSOD-Net	30.53	36.87	47.65	236.85

**Note:** ↓ indicates that a lower value is better, while ↑ indicates that a higher value is better.

**Table 5:** Results of ablation experiments on the Vaihingen and Potsdam datasets.

	L-PPM	SA-WTFD	EGA-Local	mF1 (%)		mIoU (%)		OA (%)	
				Vaihingen	Potsdam	Vaihingen	Potsdam	Vaihingen	Potsdam
Baseline	×	×	×	90.68±0.09	92.36±0.11	83.55±0.07	86.15±0.06	90.97±0.06	90.99±0.04
	✔	×	×	91.03±0.10	92.63±0.08	83.79±0.07	86.55±0.06	91.21±0.04	91.27±0.04
	×	✔	×	91.11±0.09	92.59±0.07	83.85±0.09	86.51±0.06	91.33±0.04	91.26±0.05
	×	×	✔	91.12±0.09	92.72±0.06	83.90±0.10	86.64±0.07	91.37±0.05	91.23±0.05
	✔	✔	×	91.14±0.07	92.74±0.09	83.92±0.06	86.65±0.08	91.42±0.05	91.33±0.04
	×	✔	✔	91.21±0.08	92.76±0.07	83.96±0.10	86.67±0.08	91.40±0.04	91.30±0.06
	✔	×	✔	91.19±0.08	92.77±0.07	84.01±0.09	86.69±0.07	91.44±0.06	91.35±0.05
	✔	✔	✔	91.35±0.09	92.81±0.07	84.24±0.11	86.79±0.09	91.55±0.05	91.45±0.05

**Note:** ✔ indicates that the corresponding module is included, while × indicates that it is not.

**Table 6:** Compatibility and performance analysis of different backbone networks.

Method	Backbone	Parameters (M) ↓	mIoU (%) ↑	FLOPs (G) ↓	FPS ↑
FSOD-Net	ResNet50	26.49	83.01±0.09	9.2	61.71
	ResNext50	25.96	83.25±0.08	36.46	37.33
	ResNest50	28.41	83.59±0.09	42.41	29.81
	ConvNext-Tiny	30.53	84.24±0.11	36.87	47.65

**Note:** ↓ indicates that a lower value is better, while ↑ indicates that a higher value is better.

**Table 7:** Training hyperparameter settings for each comparison model.

Method	Max_epoch_	Batch_size_	lr	Weight_decay_	Backbone_lr_
UNetFormer [43]	105	8	$6\times 10^{-4}$	0.01	$6\times 10^{-5}$
DC-Swin [42]	70	8	$1\times 10^{-3}$	$2.5\times 10^{-4}$	$1\times 10^{-4}$
BANet [41]	100	8	$3\times 10^{-4}$	$2.5\times 10^{-4}$	$3\times 10^{-4}$
A2-FPN [40]	100	4	$3\times 10^{-4}$	$1\times 10^{-4}$	$3\times 10^{-4}$
ABCNet [39]	100	4	$2\times 10^{-4}$	$2.5\times 10^{-4}$	$2\times 10^{-4}$
SparseFormer [44]	100	8	$6\times 10^{-4}$	0.01	$6\times 10^{-5}$
SFFNet [38]	105	2	$6\times 10^{-4}$	0.01	$6\times 10^{-5}$
Ours	105	2	$6\times 10^{-4}$	0.01	$6\times 10^{-5}$

**Table 8:** Class-specific F1(%) score comparison of methods on the Vaihingen dataset.

Method	ImSurf	Building	LowVeg	Tree	Car
UNetFormer [43]	93.06	95.43	85.16	90.74	88.70
DC-Swin [42]	93.23	**95.98**	85.26	90.73	88.90
BANet [41]	92.15	95.09	84.71	90.63	83.41
A2-FPN [40]	92.66	95.25	85.31	90.78	89.53
ABCNet [39]	93.11	95.40	**85.81**	90.64	87.08
SparseFormer [44]	93.26	95.85	85.28	90.61	90.21
SFFNet [38]	93.27	95.68	85.36	90.70	90.34
Ours	**93.48**	95.83	85.66	**90.92**	**90.85**

**Note:** Bold values indicate the best performance in each column.

**Table 9:** Overall performance comparison of methods on the Vaihingen dataset.

Method	mF1 (%)	mIoU (%)	OA (%)
UNetFormer [43]	90.62	83.04	91.20
DC-Swin [42]	90.82	83.39	91.41
BANet [41]	89.20	80.79	90.60
A2-FPN [40]	90.71	83.17	91.09
ABCNet [39]	90.41	82.70	91.29
SparseFormer [44]	91.04	83.75	91.33
SFFNet [38]	91.07±0.10	83.81±0.13	91.43±0.06
Ours	91.35±0.09	84.24±0.11	91.55±0.05

**Note:** Bold values indicate the best performance in each column.

**Table 10:** Class-specific F1 (%) score comparison of methods on the Potsdam dataset.

Method	ImSurf	Building	LowVeg	Tree	Car
UNetFormer [43]	92.75	95.33	86.74	88.57	96.20
DC-Swin [42]	93.56	**96.07**	87.39	89.44	96.00
BANet [41]	91.26	93.89	85.19	87.41	93.59
A2-FPN [40]	91.42	94.37	85.95	88.32	95.50
ABCNet [39]	91.53	94.25	85.87	87.17	94.86
SparseFormer [44]	93.31	95.76	87.50	89.44	96.65
SFFNet [38]	93.45	95.84	87.56	89.47	96.63
Ours	**93.87**	96.05	**87.66**	**89.69**	**96.78**

**Note:** Bold values indicate the best performance in each column.

**Table 11:** Overall performance comparison of methods on the Potsdam dataset.

Method	mF1 (%)	mIoU (%)	OA (%)
UNetFormer [43]	91.92	85.27	90.53
DC-Swin [42]	92.49	86.23	91.16
BANet [41]	90.27	82.44	88.82
A2-FPN [40]	91.11	83.88	89.46
ABCNet [39]	90.74	83.25	89.19
SparseFormer [44]	92.53	86.30	91.17
SFFNet [38]	92.59±0.08	86.41±0.09	91.20±0.05
Ours	92.81±0.07	86.79±0.09	91.45±0.05

**Note:** Bold values indicate the best performance in each column.

## Data Availability

The ISPRS Vaihingen and Potsdam datasets analyzed in this study are publicly available. The data can be accessed through the ISPRS 2D Semantic Labeling Contest benchmark portal at https://www.isprs.org/resources/datasets/benchmarks/UrbanSemLab/default.aspx (accessed on 1 September 2025).

## Abbreviations

The following abbreviations are used in this manuscript:

SFFNet	Spatial and Frequency Domain Fusion Network
CNN	Convolutional Neural Network
DWT	Discrete Wavelet Transform
EGA-Local	Edge-Guided Local Awareness Module
FFT	Fast Fourier Transform
FSOD-Net	Frequency-Spatial Feature Decoupling Network
GSD	Ground Sample Distance
HRRSI	High-Resolution Remote Sensing Imagery
ISPRS	International Society for Photogrammetry and Remote Sensing
L-PPM	Lightweight Pyramid Pooling Module
MDAF	Multiscale Dual-Representation Alignment Filter
mIoU	mean Intersection over Union
FMS	Feature Mapping Stage
R-G-B-NIR	Red, Green, Blue, Near-Infrared
SA-WTFD	Spatial Attention Wavelet Transform Feature Decomposer
